# The effectiveness of nursing interventions to improve self-care for patients with heart failure at home: a systematic review and meta-analysis

**DOI:** 10.1186/s12912-025-02867-7

**Published:** 2025-03-14

**Authors:** Jessica Longhini, Kayla Gauthier, Hanne Konradsen, Alvisa Palese, Zarina Nahar Kabir, Nana Waldréus

**Affiliations:** 1 Department of Medicine, University of Udine, Udine, Italy; 2 School of Health Studies, The University of Western Ontario, London, Canada; 3 Department of Gastroenterology, Copenhagen University Hospital—Herlev and Gentofte, Copenhagen, Denmark; 4 Department of Clinical Medicine, University of Copenhagen, Copenhagen, Denmark; 5 Department of Neurobiology, Care Sciences and Society, Karolinska Institutet, Huddinge, Sweden

**Keywords:** Heart failure, Nursing, Self-care, Home care, Transitional care, Remote care, Phone calls, Systematic review, Meta-analysis

## Abstract

**Background:**

Self-care plays an important role in the treatment of patients with heart failure (HF) and adequately performed self-care at home can contribute to fewer hospitalizations, lower mortality risk and require less emergency care. The aim of this systematic review and meta-analysis was to synthesise evidence on the effectiveness of nursing interventions on HF-related self-care at home.

**Methods:**

Medline, Scopus, Cumulative Index to Nursing and Allied Health Literature, Cochrane database, Web of Science, PsycInfo, and trial registers were searched up to 31st December 2022. We aimed to include experimental and observational studies with a control group investigating nursing interventions including transitional care, home care programs, phone calls, digital interventions, or a combination thereof on self-care of patients with HF. Outcomes were self-care maintenance, self-care management, and self-care behaviours, measured with various instruments. The screening and data extraction were performed independently by two reviewers, and disagreements were solved by a third reviewer. Cochrane risk of bias tool for randomised trials and the Grading of Recommendations, Assessment, Development, and Evaluation (GRADE) approach were used.

**Results:**

Twenty-seven studies were included (2176 participants), of which 24 were randomised controlled trials. Three categories of interventions emerged, called “transitional care”, “home care”, and “remote interventions”. Transitional care aimed at caring for patients at their homes after discharge through phone calls, digital interventions, and home visits may result in little to no difference in self-care maintenance (MD 7.26, 95% CI 5.20, 9.33) and self-care management (MD 5.02, 95% CI 1.34, 8.69) while contrasting results emerged in self-care behaviours since two out of six studies reported no improvements in self-care. Home care combined with phone calls or digital interventions likely increase self-management and self-care behaviours (MD -7.91, 95% CI -9.29, -6.54). Remote care could improve self-care behaviours when delivered as phone call programs, but they are ineffective on all outcomes when delivered as digital interventions alone.

**Conclusion:**

Transitional care and home care combined with phone calls and digital interventions, and phone calls caring for patients at their home could slightly improve self-care in patients with HF. However, more research is needed to study the effects across different domains of self-care and of interventions delivered through digital interventions alone.

**Supplementary Information:**

The online version contains supplementary material available at 10.1186/s12912-025-02867-7.

## Introduction

Heart failure (HF) is defined as a complex clinical syndrome consisting of typical symptoms and signs [1]. In the adult population, the prevalence of HF ranges between 1% and 3% and the hospitalization rates due to HF are estimated to be 1–2% of all hospital admissions [1, 2]. Among patients with HF, older adults aged ≥ 65 years have the highest hospitalisation rates and the poorest outcomes with a high risk of death at 1 year [2, 3]. In addition, evidence reported that the risk of 90-day unplanned readmission could be significantly increased by frailty [4]. A global investigation showed that the 30-day readmission rate was 13% (median age 76.8 years) and a 1-year readmission rate was 36% (median age 77.2 years) [5].

As part of a prevention strategy, current guidelines recommend a multidisciplinary approach, including self-care education as part of HF treatment [1, 6]. Patients with HF who perform adequate self-care have fewer hospitalisations, a lower mortality risk, and require less emergency care [6, 7]. Self-care is defined as “the process of managing health through health promoting activities and managing illness” [8]. Practicing self-care includes adhering to appropriate maintenance behaviours (e.g., healthy diet, medication adherence), monitoring behaviours (e.g., weighing daily), and management behaviours (responding to changes in signs and symptoms) [9]. Self-care is a particularly vital aspect of care when patients transition from the hospital to their home and when they are stable to preserve clinical stability. Therefore, individuals need to manage HF in their daily lives at home to prevent symptoms and re-hospitalisation [1, 6, 7], however, many struggle with consistent adherence to self-care, leading to the most frequent clinical events [7].

Numerous studies have reported inadequate self-care levels in HF patients, especially among older adults [10, 11]. Inadequate self-care in HF is a complex phenomenon since several factors can affect it, such as self-care efficacy, self-care confidence, HF knowledge, health literacy, number of symptoms, patient activation levels, cognitive function, perceptions about the barriers to self-care, caregiver relationship, quality of social support, employment income, and prefrontal brain tissue integrity [12]. Therefore, the need and the effort to learn performing adequate self-care are significant among adults, and especially among the elderly who could be affected by deficits in the ability to detect sensations arising within the body and in cognition to monitor, interpret and manage symptoms [13, 14].

Therefore, promoting self-care in patients with HF is challenging for healthcare providers, also because it takes time for patients to change their behaviours. Promoting self-care implies the implementation of multidimensional and complex interventions including motivational support and behavioural change techniques, such as goal setting and self-monitoring, educating patients to provide them with the knowledge and skills needed to manage their conditions, such as medication management or dietary planning, creating a supportive environment, including family involvement and access to community resources [15]. Moreover, implementing effective models of care to allow healthcare providers sufficient time to implement complex interventions to promote self-care also requires time. This process can be further hindered by patients’ difficulties in physically getting to health services [16, 17]. Patients may often need home care due to barriers that prevent their access to public services and resources, such as living in a remote area, disability, lack of welfare or social support and lack of accessible public and private transport [10, 11]. Older adults, representing a vulnerable population at higher risk of HF readmission [2, 3], experience these barriers more frequently than younger people, which results in greater difficulties in performing adequate self-care [11, 14]. Thus, older adults have a greater need for self-care education than younger people. Therefore, fostering models of care that allow patients’ care needs to be met at home care by developing and strengthening their self-care abilities is needed to ensure proper and equal care for patients struggling to manage their HF due to inequitable barriers. In addition,

Nurses play an important role in promoting self-care in patients with HF by educating them about how to prevent possible complications and readmissions [18], both in nurse-led interventions and in interventions delivered as multidisciplinary care guided by other professionals, where nurses cover an important role for clinical monitoring and patient education. Nurse-led interventions are described in several previous systematic reviews and are the activities planned and directed by the nurse, whereas nursing interventions are activities within a nurse’s scope of theory and practice aimed at alleviating patient suffering and promoting health. Therefore, considering the difficulties HF patients face in learning self-care, nursing interventions delivered at home to promote self-care are important both in the period after hospitalization and periods when patients are stable and do not experience hospitalization since patients need time to learn all self-care behaviours and implement them daily [8, 19]. Patients in the post-hospitalization learn self-care abilities to preserve safety and avoid new exacerbations. However, they often need several months beyond the initial period post-hospitalization phase, to fully adapt to a new diagnosis or an acute event. This time is essential for them to acquire the necessary knowledge and develop the confidence needed to manage their condition effectively. During this period, patients learn to interpret their symptoms and identify solutions to underlying issues through a reflective process. This process enables them to make informed and contextualized decisions regarding their health and cultivate self-regulation skills, which are essential for implementing chosen solutions effectively, adopt and maintain behavioural changes [8, 19]. Therefore, interventions can be partially delivered, as part of transitional care programs, or totally delivered at home and include different components. For example, transitional care could take place after hospital discharge at home through, telephone follow-up, home visits, and remote care includg telemonitoring, mobile apps and web-based interventions. The same modalities can be applied in at-home interventions that occur independently of hospitalization. These interventions help facilitate patient care, ensure continuity of care, and promote patient engagement in developing self-care behaviours. Additionally, home visits are valuable for involving informal caregivers, families, and social networks in contributing to self-care. They also allow for the evaluation of the living environment to identify and address barriers to self-care that are beyond the patient’s control. In addition, the different modalities could be used to perform educational interventions through motivational interviewing and messages, written materials, digital educational materials through web-based solutions, and mobile apps that serve as instruments to promote and foster self-care by improving knowledge, attitudes, self-efficacy, and motivation.

Previous reviews have shown that nurse-led education interventions reduce readmission and mortality due to HF [20] and promote self-care among patients with HF [21]. However, available reviews have been conducted mixing hospital, outpatient care, and home care [21] or have focused on specific interventions such as motivational interviewing [22] or mobile apps [23], or telecoaching [24], thus an overview of interventions that can be delivered at the patients’ home, from the post-hospitalization phase to a more stable phase independent from hospitalization, is missing. Other limitations include that reviews are not directed toward HF specifically [25] and self-care related outcomes [24]. In addition, an available review focused exclusively on self-care measured with Self-Care of Heart Failure Index, excluding the other self-care measures [21]. To date, there is no summary of nursing interventions targeting self-care improvement where the patient can stay at home without reaching physically a health service, making it challenging to draw conclusions about the effectiveness on self- care for patients with HF.

## Aim

The aim of this systematic review and meta-analysis was to synthesise evidence on the effectiveness of nursing interventions on HF-related self-care at home.

## Methods

### Design

This systematic review with meta-analysis was conducted under the guidance of the registered protocol (PROSPERO *blinded for reviewers*) and Cochrane Handbook for Systematic Reviews of Interventions, and reported according to the Preferred Reporting Items for Systematic Reviews and Meta-Analyses (PRISMA) checklist [26] (Additional File 1).

### Inclusion and exclusion criteria

Inclusion criteria were as follows (i) Studies: randomised controlled trials (RCTs), quasi-experimental studies, and observational studies with a control group, according to their wide diffusion in primary settings to inform on interventions’ efficacy [27]; (ii) Patients: living in the community, aged over 18 years, with HF in any New York Heart Association (NYHA) class; (iii) Intervention: delivered by nurses (placed in different services such as general practice, home care and telemonitoring services, hospital with transitional care programs, outpatients clinics) at patient´s home, including interventions delivered partially at home, defined as transitional care when post-hospitalization follow-ups do not require patients to reach outpatient clinics or the hospital, covering different phases of the HF condition; and (iv) Outcome: HF-related self-care measured quantitatively with validated instruments. Exclusion criteria were studies that were conducted completely in the hospital, intermediate care, long-term care, and residential settings. Self-care confidence was not considered a suitable outcome, since it is not a self-care behaviour, but rather a powerful influencing factor on self-care [28]. Only articles in English were eligible for inclusion.

### Literature search and study selection

Two authors independently (JL, KG) searched Medline, Cochrane Library, Web of Science, Scopus, PsycInfo, and Cumulative Index to Nursing and Allied Health Literature in close collaboration with a librarian, from database start date until December 31, 2022. The results of the searches from the two authors were compared to find any differences. No differences emerged. We combined terms related to HF, nursing, and self-care with Boolean operators in search strings (Additional file 2).

### Screening process and data extraction

All studies were assigned to two authors (JL, KG) who independently screened the titles and the abstracts. In a second step, authors screened the full text, for which the level of agreement was 0.82 (Cohen’s kappa, Confidence Interval [CI] 95% 0.70–0.91), demonstrating good inter-rater reliability [29]. Disagreements were resolved by a third author. The screening process was managed on the online platform Rayyan.

Two authors (JL, KG) independently extracted data regarding the first author, design, country, inclusion criteria, population characteristics, intervention characteristics, outcomes measured (types, values at each follow-up available), and related assessment instruments in each of the included studies. An Excel spreadsheet was used to organise and track the data. No disagreements were found when the two independent extractions were compared by a third author. Corresponding authors of the included studies were contacted for clarification when data were missing.

### Quality and certainty of the evidence assessment

Five authors performed the quality assessment (JL, KG, HK, ZNK, NW). Two authors at a time independently performed a risk of bias assessment with the revised Cochrane risk of bias tool for randomised trials (RoB 2) [30], and the risk of bias tool for non-randomised studies of Interventions (ROBINS-I) [31]. A third author resolved disagreements. The certainty of evidence was evaluated by the Grading of Recommendations, Assessment, Development, and Evaluation (GRADE) approach for each outcome category [32].

### Outcome

The main outcome was the patients’ self-care related to HF measured with a validated instrument in any language. The results from studies were grouped into three categories according to the instrument used to measure self-care. Studies that utilized the Self-Care of Heart Failure Index (SCHFI) [33] to assess self-care were categorized under ‘self-care maintenance’ (live a healthy life style, adhere to treatment, monitor symptoms) and ‘self-care management’ (recognizing and evaluating changes in health, deciding to take action, implementing treatment strategy, evaluating the treatment implemented) aligned with the respective scales of the instrument. Studies adopting for example the European Heart Failure Self Care Behaviour questionnaire (EHFScBs) [34, 35] were included in a third category, called ‘self-care behaviours’ (maintaining health through health-promoting activities and by managing illness) [8].

### Data analysis

Interventions were grouped in three categories called (a) Transitional care, including interventions with face-to-face sessions during hospitalization and post-discharge care delivered at home through home visits, phone calls, or digital interventions or a combination thereof, (b) Home care, including interventions with home visits combined with phone calls or digital interventions, and (c) Remote care, including interventions with phone calls or digital interventions alone.

A meta-analysis was conducted by pooling studies in the same intervention category, deemed homogeneous in population characteristics, and using the same questionnaire with the same items and range of responses to allow a direct comparison in the meta-analysis. Studies were not included in the meta-analysis if they did not provide sufficient data that we were not able to obtain from the original authors, or investigated interventions not grouped in the same category (i.e. transitional care), or populations with characteristics different from other studies included in the metanalysis (i.e. higher prevalence of patients in NYHA class IV), or used different instruments or different versions of the instrument because we deemed it important to provide an effect size on precise behaviours. We calculated the mean difference (MD) as effect size. The random-effects model was used, considering the heterogeneity among interventions’ components detected during the analysis of study characteristics [36]. Heterogeneity was assessed via the I^2^ statistic; a value of 30–60% was considered moderate heterogeneity, while values greater than 75% marked considerable heterogeneity [37]. Quasi-experimental studies were included in the meta-analysis only if they were judged to be at low or moderate risk of bias [37].

We performed a sensitivity analysis to check the robustness of the results for each outcome category by removing quasi-experimental studies and studies at high risk of bias. Small studies bias was investigated through the visual inspection of the funnel plots when at least 10 studies were available. Analysis was performed with Review Manager 5.4 [38] and R Software with the package *metafor* [39]. When a meta-analysis was not possible, results were summarised narratively.

## Results

### Description of studies

**Fig. 1:**
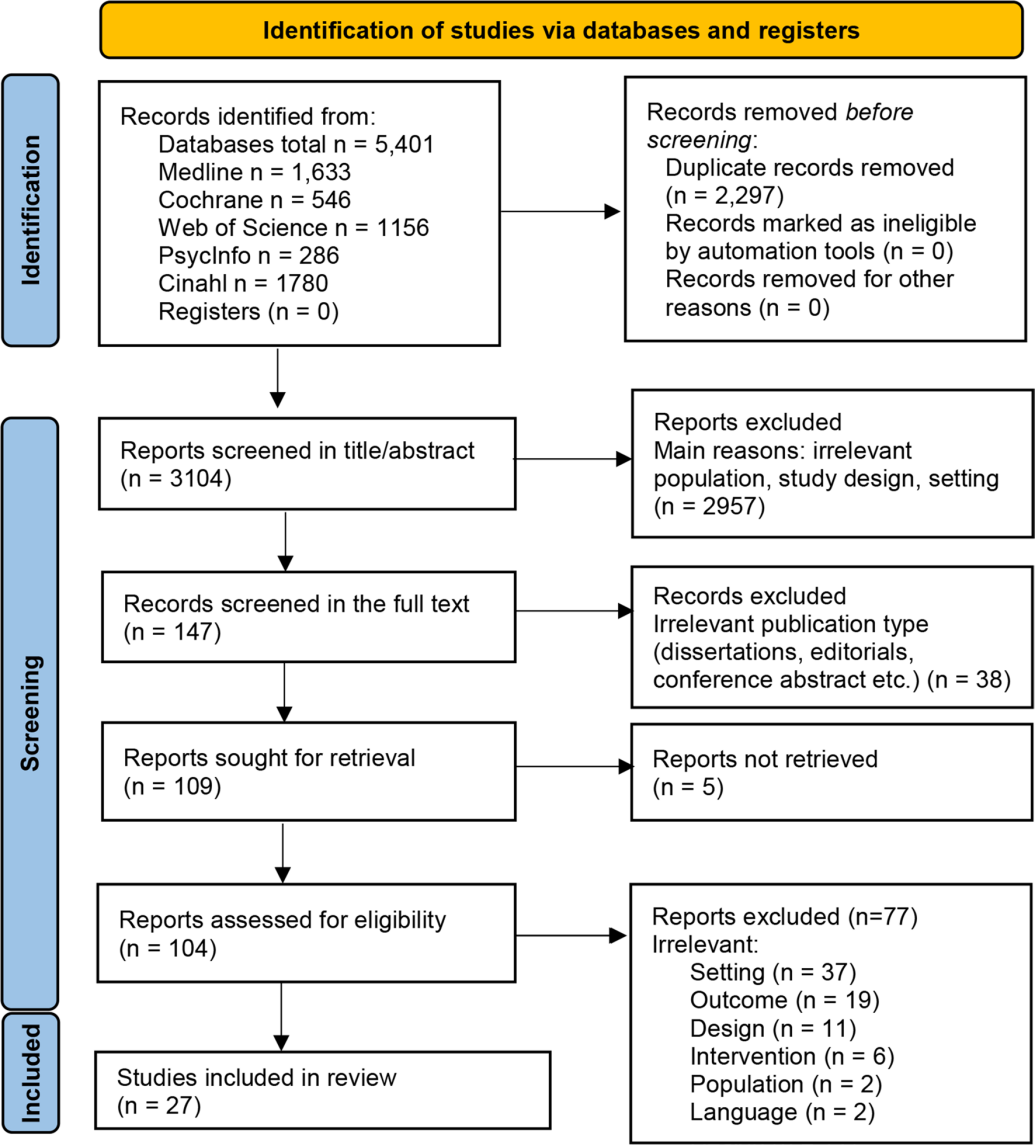
PRISMA 2020 flow diagram for new systematic reviews which included searches of databases and registers only (Page et al., 2021). **Legend**. Cinahl, Cumulative Index to Nursing and Allied Health Literature

We retrieved 5401 studies from databases and selected 3104 studies after removing duplicates that were screened by the title and abstract screening. The full-text screening was performed for 147 studies, of which 76 studies were excluded (Additional File 3), and, finally, 27 studies were included [40–66] (Fig. 1). The studies were published between 2000 and 2022, and 24 were RCTs, and three were quasi-experimental studies. No observational studies with a control group were detected. Most studies were conducted in the United States of America (*n* = 9) (Table 1). Across the studies, the sample size ranged from 20 [41] to 252 [46] with a total of 2176 participants across all included studies.

**Table 1:** Characteristics of studies included

Last Name of the first Author, year Country Study design	Setting of Recruitment Setting of intervention	Population	NYHA class Mean EF% at the baseline (SD/IQR 95%)	Intervention HCPs Delivering interventions	Control group/comparison	Sample size at baselines, dropout rate at follow-up	Outcome measures	Data collection instrument /tool (Reference of original tool)
**Transitional care with face-to-face sessions, home visits, phone calls, or digital contact**								
Balk 2008 Netherlands RCT	Hospitals Hospital + TV channel + digital intervention	Patients with CHF in stable condition + NYHA class I-IV 66 years (33–87)	7% of patients in NYHA class I, 40% of patients in NYHA class II, 48% of patients in NYHA class III, 2% of patients in NYHA class IV 31 (9–71)	Personalized plan (prescribed medication, advice about salt restriction, fluid intake, and lifestyle regimen) + MOTIVA system: band home TV-channel with educational material, health related surveys, motivational messages for lifestyle, reminders of medication. Intervention-plus: MOTIVAS + automated devices for daily measurements of blood pressure and weight + ranges were for blood pressure and weight + MSC staff available during office hours for phone contacts and analysis of the daily measurements Medical Service Center nurses trained in heart failure management	Managed by cardiologists and HF-nurses as standard local practice	214; Follow-up from 2 to 537 days (mean 288 days)	Self-care behavior	European Heart Failure Self Care Behavior Scale (Jaarsma 2003)
Chen 2018 China RCT	Hospital cardiology department Hospital + phone calls	Hospitalized patients with CHF aged 18–70 years 59.9 years (6.93)	8% of patients in NYHA class I, 43.5% of patients in NYHA class II, and 48.5% of patients in NYHA class III NA	4 MI sessions during hospitalization (15–20 min) to plan behavioural changes + telephone follow-up at 2, 4, 8 weeks (10–15 min) on behaviours on the health status, life, work, difficulties during changing behaviours; providing professional suggestions and reinforcing commitment to complete self-care plans. Investigator highly experienced in MI and cardiovascular nursing	3–4 health education session by a nurse during hospitalization, telephone follow-up as intervention group	72; 2 months: 13.9%	Self-care maintenance and management	Self-Care of Heart Failure Index (Riegel 2009)
Cossette 2016 Canada RCT	Hospital Hospital + phone calls	HF patients who live with a primary caregiver with no cognitive problems and no planned regular specialized follow-up, dyads included 67 (NA)	1 patient in NYHA class I, 13 patients in NYHA class II, 15 patients in NYHA class III, and 3 patients in NYHA class IV *calculated*: 30.62 (12.55)	5 sessions: 2 of 30–45 min during hospitalization (1 with the dyad and 1 only with caregiver) + 3 phone calls (10 min) Intervention guide integrating Self-Determination Theory elements (perceived competence, autonomous motivation, and perceived relatedness). Role-playing involving the nurse and the caregiver during the encounters to provide practice in autonomy-supportive behaviors relating to patient self-care + educational checklist for nurses Project nurse	Usual discharge planning, bedside nurse providing information on HF, medication, nutrition. No telephone or hospital follow-up.	32 patient-caregiver dyads; 1 month: 15.6%	Self-care maintenance	Self-Care of Heart Failure Index - (Riegel 2009)
Davis 2012 USA RCT	Academic hospital Hospital + phone call	Patients hospitalized with exacerbation of heart failure and mild cognitive impairment 59 (13)	2.4% of patients in NYHA class I, 45.6% of patients in NYHA class II, and 47.2% of patients in NYHA class III, 4.8% of patients in NYHA class IV 34 (19)	Self-care teaching (mean 44 min) based on cognitive training and environmental manipulation during hospitalization: workbook with pictograms for self-care schedule, personalized plan, problem-solving with scenario, audiotaping of teaching sessions, medication organizer and daily weight demonstration, self-monitoring diary, measuring cups + phone call at 24–72 h with teach back Nurse case manager	Standard discharge teaching for HF with HF booklet on symptom recognition and management, exercise, medication adherence, dietary and fluid restrictions,	125; 1 month: 12.8%	Self-care-maintanance and management	Self-Care of Heart Failure Index (Riegel 2009)
Evangelista 2015 USA Quasi-experimental study	Hospital Hospital + digital intervention + phone calls	Patients hospitalized with acute exacerbation of heart failure with no dementia or serious comorbidities 72.7 (8.9)	69% patients in NYHA class II, and 30.9% in NYHA class III 26.5 (6.4)	Remote monitoring system platform, teaching to take weight, heart rate, and blood pressure daily for 3 months, step-by-step guide, number available 24/7. Phone call 24–48 h after discharge, teleconferencing, nurse- primary care provider collaboration to set limited advice, reset thresholds, provider outpatient visit, ER evaluation Research nurse	Primary care and specialty visits, home healthcare, post-hospital outpatient visits, 1 nurse phone call 1 day after discharge	42; 3 months: 0%	Self-care maintenance and management	Self-Care of Heart Failure Index (Riegel 2009)
Hoover 2017 USA Quasi-experimental study	Hospital Hospital + home + phone calls	Individuals ≥ 21 years able to read and understand English with no new diagnosis of HF and no significant cognitive impairment. 76.4 (range: 50–96)	62% NYHA class III-IV NA	Coleman Care Transitions Intervention: Education/pharmacist medication reconciliation and teaching from nurses and pharmacist with patients and caregivers during hospitalization + 1 home visits within 72 h of discharge + 3 phone calls over the remaining 4 weeks focused on medication awareness and self-management, personal health record, appointments, signs and symptoms of exacerbations. Registered Nurse Transition Coach	Evidence-based HF education and medication reconciliation at the discharge + follow-up visit within 2 weeks by the provider or specialist.	71; 1 month:7%	Self-care Maintenance and management	Self-Care of Heart Failure Index (Ref. NA)
Hwang 2022 South Korea RCT	Hospital Hospital + phone calls	Patients hospitalized with heart failure with no cognitive impairment or serious comorbidity 66.2 years (13.58)	9.8% patients NYHA class I, 19.7% in NYHA class II, 32% in NYHA class III, and 38.5% in NYHA class IV 40.30 (14.23)	1 educational session during hospitalization (1 h) with written materials on HF signs & symptoms, treatment, diet and exercise recommendations and teach-back + 3 phone calls at 2, 4, and 8 weeks after discharge to manage barriers to self-care Principal Investigator - Nurse	Pre-discharge care to review medication and schedule follow-up appointment	122; 3 months, 6 months: 18%	Self-care behaviors	9-item European Heart Failure Self-Care Behavior Scale (Jaarsma 2009)
Jaarsma 2000, Netherlands RCT	Hospital Hospital + phone call + home visit	Patients hospitalized with symptoms of chronic heart failure, over the age of 50, NYHA l class III and IV; heart failure diagnosis for longer than 3 months 74 years (9)	17% of patients in NYHA class III, 22% in NYHA class III-IV, and 61% in NYHA class IV 34 (14)	Patient and family education in 4 sessions during hospitalization, a card on morning symptoms, a phone call within 3 days after discharge, and a home visit within 10 days after discharge for monitoring and teaching on disease and contacting a cardiologist, GP, or ER in case of problems. Education also includes HF self-care, medication adherence, support to access services, setting priorities, transports, etc. Educational materials on diet and fluid restrictions. Study nurses	Not structured patient education, a follow-up phone call, or a home visit from a nurse	186; 1 month, 3 months, 9 months: 29%	Self-care abilities; Self-care behavior	Heart Failure Self-Care Behavior Scale
Koberich 2015 Germany	Cardiology department of heart failure clinic Hospital + phone calls	Patients with LVEF </=40%, NYHA II - IV 61.7 years (12)	66.4% of patients in NYHA class II, 29.1% of patients in NYHA class III, 4.5% of patients in NYHA class IV NA	1 standardized education session on HF and HF self-care during hospitalization or at the clinic + 4 phone calls within three months (1, 4, 8 and 12 weeks after discharge or outpatient clinic appointment). Education on HF, medications, lifestyle, signs and symptoms and their management, traveling and recreational activities. Educational booklet on HF + diary for parameters. Nurse, principal investigator	Standard medical treatment	128; 3 months: 14.6%	Self-care behaviors	9-item European Heart Failure Self-care Behavior Scale (Jaarsma 2009)
Leavitt 2020 USA RCT	Hospital Hospital + home visit	Patients with primary or secondary diagnosis of heart failure, over age 65, with no dementia 82.7 years (8.27)	NA 2 patients 15–19%, 5 patients 20–29%, 3 patients 30–39%, 5 patients 40–49%, 18 patients 50–59%, 6 patients 60–69%, 1 patient 70–79%	1 visit during hospitalization providing a HF education booklet, HF teaching on pathophysiology, symptoms, and medications + 4 home visits over 30-days (1 within 72 h after discharge) for physical assessment, examining home environment, daily-life HF coping strategies CareNavRN nurse: RN interested in cardiology and at least 2 years of experience in home health	Printed and verbal HF discharge education delivered by primary hospital RN + home health care. No visits by the CareNavRN nurse.	40; 1 month: 0%	Self-care Maintenance and management	Self-Care of Heart Failure Index (Riegel 2009)
Moon 2018 Republic of Korea Quasi-experimental study	Hospital Hospital + phone calls	Patients with heart failure diagnosis of at least 6 months, LV EF < 50%, NYHA class II/III 13 (34.2%) patients aged 60–64, 7 (18.4%) age 65–69, 18 (47.4%) age 70–75	NA 40.06 (6.57)	Education session on HF, the disease status, and self-management during hospitalization + educational booklet on HF, self-reported symptoms, self-care behaviours + phone calls (15–30 min) 1 time/week for 4 weeks on symptoms, problems with self-management, HF education on lifestyles, stress management Nurse	NA	42; 5 weeks: 9.5%	Self-care behavior	9-item European Heart Failure Self-care Behavior Scale (Jaarsma 2009)
Sadeghi Akbari 2019 Iran RCT	Heart Center Hospital + phone calls	Patients with heart failure, an ejection fraction of less than 40, NYHA class II and III, no cognitive or psychiatric disorder or comorbidity *calculated*: 68.6	NA < 40% (no mean reported)	Illness perception correction-based education (30 min per session) based on Leventhal self-regulation model over 3 days during hospitalization (30 min) on HF and control ability, written and verbal education on medications + an educational booklet + 8 weeks of phone calls (10 min) 1 time/week. Research nurse	Routine nursing care, including education on medications and advice to limit daily salt and water, weigh themselves daily and other advice related to their disease	76; 2 months: 7.9%	Self-care behaviors	European Heart Failure Self-care Behavior Scale − 12 items
Sun 2019 China RCT	Hospital Hospital + internet based medical platform + phone calls	Patients with CHF NYHA class > II IG: 68.21 (4.69) CG: 68.57 (4.12)	35% patients in NYHA class II, 49% in class III, and 16% in class IV NA	Personalized health education plan with daily health education content on HF knowledge, treatment plan and goals, medication, exercise, nutrition, and prevention of acute attack over 3 days to help patients correct their self-care plan and monitored its implementation during hospitalization. After discharge: internet-based medical platform with a nurse available 9–17 daily + weekly health education program + self-care plan + messages sent with APP platform or WeChat public account + weekly phone calls for 3 months, 1 time/every 2 weeks after 3–4 months, 1 time/months after 5–6 months. Meeting of research team members every 2 months where patients or families were invited. Cardiology nurse, nursing staff	Nursing guidance at discharge with a health education manual + phone calls 2 weeks after discharge for monitoring and health guidance	100; 3 and 6 months: 0%	Self-care maintenance and management	Self-Care of Heart Failure Index (Riegel 2009)
Wonggom 2020 Australia RCT	HF outpatient clinic in hospital Hospital ward/HF clinic + m-health at home	Patients with a diagnosis of HF, NYHA class I to IV	50% of patients in NYHA class I, 41.7% of patients in NYHA class II, 8.3% of patients in NYHA class III, 0% of patients in NYHA class IV 33.3% of patients with a LVEF < 40%, 30.6% of patients with a LVEF 40–49%, 36.1% of patients with a LVEF ≥ 50%	1 session to teach how to use the app during hospitalization + avatar app on tablet on the topic: understanding HF, looking after yourself, things to do every day, emergency action plan. Research nurse	Bedside education, follow up at the HF clinic, booklet	36; 1 month, 3 months: 2,8%	Self-care maintenance and management	Self-Care of Heart Failure Index (Riegel 2009)
Yu 2015 China RCT	Hospital Hospital + home visit + phone calls	Patients with chronic health failure + >/= 60 years IG: 78.6 (7.1); CG: 78.7 (6.7)	57.9% patients in NYHA class II, 39.3% in class III, and 2.8% in class IV IG:41.1 (16.1); CG: 39 (9.3)	Health counselling through pre-discharge visits, home visits on health status, self-care, concerns, beliefs + 2 weekly home visits focused on HF, psychological issues, educational on monitoring and management and self-care goals, find community support services + intensive telephone follow-up to monitor symptoms, give advice on self-care, counselling on the action plan, barriers to self-care + telephone access to cardiac nurse; Phone calls 1 week after the second home visit, then every 2 weeks for 3 months, and then every 2 months for 6 months. Cardiac nurse with a professional diploma in cardiovascular nursing and > 10 years of clinical experience in cardiac care	Teaching from pharmacy dispensers on hospital discharge, visits at the specialist clinic 4/6 weeks after discharge.	178; 6 weeks: 12.9%, 3 months: 19.7%, 9 months:32.6%	Self-care maintenance and management	18-item Chinese version of the Self-Care Heart Failure Index (modified version of Riegel 2004)
Zamanzadeh 2013 Iran RCT	Hospital Hospital + phone calls	Patients diagnosed with NYHA class III or IV HF, had an LVEF < 40% 63.5 years (NA)	48.7% in class III, and 51.3% in class IV IG:25.73 (9.2) CG: 24.05 (8.94)	HF education session (1 h) during hospitalization, individualized education booklet, phone calls (15 min) every two weeks for 3 months after discharge to check signs and symptoms and self-care behaviours. Education on signs and symptoms, medications, self-care behaviours, and lifestyles (i.e. diet). Based on Orem’s self-care theory. Nurse	Usual care provided by the hospital and physician visits	80; 1 month, 2 months, 3 months: 2.5%	Self-care maintenance and management	Self-Care of Heart Failure Index (Riegel 2009)
**Home care with digital interventions or phone calls**								
De Souza 2014 Brazil RCT	Hospital Home + phone calls	Patients hospitalized for acute decompensated heart failure with LVEF < 45% 62 (13)	6.3% of patients in NYHA class I, 37.6% of patients in NYHA class II, and 46% of patients in NYHA class III, 9.9% of patients in NYHA class IV 29.6 (8.9)	4 home visits at 10–30 -60–120 days after discharge (60 min) for physical examination and education + phone calls (10 min). Education was on disease, self-care, medication adherence, side effects, signs and symptoms of exacerbation. Nurses	Outpatient visits with GP	252; 6 months: 3.6%	Self-care	European heart failure self-care behavior scale (Jaarsma 2003)
Clark 2015 USA RCT	Physician/advanced practice registered nurse (APRN) referrals, HF clinics, and media Home + phone calls/ e-mail	Non-hospitalized patients with chronic HF and NYHA class I to III; 45 years or older; living at home independently 62.4 (10.9)	14% of patients in NYHA class I, 42% of patients in NYHA class II, 44% of patients in NYHA class III NA	Educational and skill-building program based on Stuifbergen’s health promotion focused on self-efficacy, Improving Your Memory booklet: 3 months of home visits every 10 to 14 days (1–1.5 h) for a total of 8 sessions on lifestyles, sign and symptoms, medications, stress, social and intimate relationships + 3 months of phone calls and/or e-mail (5–15 min) + 3-months no home visits, e-mails, or phone calls but only communicating with their physician if questions APRNs who were adult clinical nurse specialists with master’s or PhD education and expertise in HF and advanced cardiovascular nursing	Information on health promotion for adults/older adults with a notebook without HF information, scheduled meetings, no phone calls and e-mail	50; 6 months: 0%	Self-care maintenance and management	15-item Revised Selfcare of Heart Failure Index (Riegel 2004)
Jiang 2021 Singapore 3-arm RCT	Hospital Home + digital intervention	Patients with HF using a smartphone everyday CG: 68.82 (13.14); IG A: 69.08 (10.51); IG B: 66.82 (11.81)	I-II (Mild to Moderate) NYHA class: CG: 30.4%; IG A 32.7%; IG B 28.1% NA	Usual care + group A and group B. Both group A and B received the HOM-HEMP intervention as HF self-management program adopting a psychosocial education approach with HF self-management toolkit + 3 home visits (40 min–1 h; 1 every 2 weeks). Educational materials on HF, salty foods, + drinking mugs with marks, scale, pill box with alarms + Educational plan + Motivational interviewing. Group B received a smartphone app with reminders for medication and appointments, weight/blood pressure/ symptom logs, educational information, chat room with nurse Research nurse trained in MI with 10 years of clinical experience	Medical, nursing, allied health and follow-up services at the hospital	213 randomized/ 177 at the baseline; 3 months from baseline 4.5%, 6 months from baseline 8.5%, 6 months from randomization 23.9%	Self-care maintenance and management	Self-care of heart failure index (Riegel 2009)
Hoban 2013 USA RCT	No profit home healthcare agencies Home + digital intervention	Patients a primary diagnosis of HF in the agency data enrolled in the HHA for home care services. 78.4 years (NA)	NA NA	Same as control group + telemonitoring equipment. Data were monitored by a telemonitor nurse coordinator daily or more frequently who contacted the patient, primary nurse, or physician when any changes in or missing data occurred. The telemonitor nurse coordinator and cardiac nursing team (experienced home healthcare registered nurses with a strong cardiac background)	Home visits 2–3 times/week, teaching on HF medications, low-sodium diet, fluid restrictions, daily weights, physical activity, HF booklet with symptoms listed and diary for weights	80; 3 months: 25%	Self-care behavior	Self-Care of Heart Failure Index (Riegel 2004)
Masterson Creber 2016 USA RCT	Hospital Home + phone calls	Patients hospitalized with primary or secondary diagnosis of heart failure, class NYHA II-IV, with no psychosis or cognitive impairment 62 (13.4)	16.4% patients in NYHA class I/II, and 83.6% in class III/IV 36 (18.14)	Home-based MI to identify at least 2 personalized goals related to HF self-care, personalized plan reinforced with 3–4 phone calls over 90 days. Nurse	Patient education materials to help identify and address self-care barriers, maintain a lower sodium diet, active lifestyle targeting goal behavior changes through participant interaction	100; 3 months:33%	Self-care Maintenance	Self-Care of Heart Failure Index (Riegel 2009)
Motta 2013 Brazil RCT	Referral centers for HF patient treatment Home + phone calls	HF with ejection fraction of 45% or less, hospitalized due to decompensation of the disease n IG was 62.49 ± 13.65, against 63.37 ± 12.05 in CG	6.5% of patients in NYHA class I, 41% of patients in NYHA class II, and 40.5% of patients in NYHA class III, 10.5% of patients in NYHA class IV IG: 29.3 ± 8; CG: 30.3 ± 9.5	Home visits at 10th day, 1-2-3 months after discharge according to a protocol (Education on medication, weight control, salt and fluid restriction, physical exercise, annual vaccination, symptom recognition) + 4 phone calls 15 to 30 days after the visits Nurses specialized in HF patient care	Usual care with outpatient visits or not	200; 6 months: 24.5%	Self-care; Treatment adherence	European Heart Failure Self Care Behavior Scale − 12 Items;
Shao 2013 Taiwan RCT	Cardiac clinics Home + Phone calls	patients with HF + >/=65 years + NYHA I - III + discharged from cardiology wards 72.04 (5.48)	7.4% patients in NYHA class I, 65.7% in class II, 26.9% in class III < 20%: 7.4% of patients; 21–40%: 70.4% of patients; >40%: 26.9%of patients	Self-management program focused on self-efficacy (Bandura 1997). The program consisted of 5 sessions of self-management program (1 home visit within 3 days after enrolment, 4 phone calls at 1, 3, 7, 11 weeks), diary of daily sodium and fluid intake, daily weight. Education on HF, self-monitoring and self-management. Action plan to monitor low salt diet, fluid and weight. Nurse	Phone calls at 3, 7, 11 weeks after discharge from the research assistant using the ‘Telephone guide-control group’, different from the intervention group.	108; 1 month and 3 months: 13.9%	Self-management	European Heart Failure Self-care Behavior Scale (Jaarsma 2003) - modified version: Heart failure Self-management behavior scale − 10 items
**Remote care with digital interventions or phone calls**								
Brandon 2009 USA RCT	HF DRG list provided by the cardiologist Phone calls	Adults with HF IG: 60 years (49–69); CG NA	25% of patients in NYHA class I, 50% of patients in NYHA class II, 20% of patients in NYHA class III, 5% of patients in NYHA class IV NA	7 calls delivered as 1 call/week for 2 weeks, then every 2 weeks for 10 weeks; education on HF, low sodium diet, calling physician with symptoms of exacerbation, smoking cessation, flu/pneumonia vaccinations, medication adherence Advanced practice nurse	Education from physician/RN on exercise, low sodium intake, medication, calling physician with symptoms of exacerbation, variable frequency of clinic visits	20; 3 months: 0%	Self-care behavior	Revised Self-care behaviors scale (Artinian 2002)
Shearer 2007 USA RCT	Hospital Phone calls	Patients with HF over 21 years of age 76.03 (8.32)	41.9% patients in NYHA class II, 47.7% in class III, 8.1% in class IV; 2.3% NA IG: 36% (14.86); CG: 33% (15.76)	Same as control group + telephone-delivered empowerment intervention with phone calls 1 to 3 days after discharge, then at 2, 4, 6, 8, 12 weeks on valued goals, HF signs and symptoms, adherence to an action plan on self-care, self-management, and functional health. Base on Rogers’ Science of Unitary Human Beings person-environment process to foster empowerment. Calls were conducted according to a standardized script. Nurse clinicians experienced in HF care	Nursing standardized HF education plan on HF signs and symptoms and plan care adherence to a prescribed plan of care during hospitalization	71; 3 months:8.4%	Self-management	Self-Management of Heart Failure (SMHF) scale
Ware 2022 Canada RCT	Heart Function Clinic in hospital Digital intervention	Patients with diagnosed with HF with reduced ejection fraction (< 40%), uncontrolled HT (≥ 140/90 mm Hg auscultatory), or insulin-requiring DM and performing self-capillary glucose monitoring 59 (12.6) for overall sample not only those with HF	NA NA	Smartphone + Bluetooth devices (weight scale, blood pressure monitor, and blood glucose monitor); instruction to monitor their daily weight, blood pressure, heart rate, and symptoms, and Medly smartphone app. The app was for (a) telemonitoring to record physiological measurements with wireless home medical devices, (b) receiving automated answer symptom questions and self-care instructions based on algorithms (the app send alerts to the clinical team via email). Historical trends were viewable on a secure web portal. None or nurse practitioner	Clinical visits every 3 to 6 months, optimization of medical therapy, self-management education	66; 6 months: NA for HF	Self-care maintenance and management	Self-Care of Heart Failure Index (Riegel 2009)
Vuorinen 2014 Finland RCT	Cardiology Outpatient Clinic of Helsinki University Central Hospital Digital intervention	Patients with heart failure, with age of 18–90 years, NYHA class ≥ 2, LVEF ≤ 35%, need for a regular check-up visit, and time from the last visit of less than 6 months CG:57.9 (11.9); IG:58.3 (11.6)	38.3% of patients in NYHA class II, 58.8% of patients in NYHA class III, 3.2% of patients in NYHA class IV CG:28.6 (5.0); IG:27.3 (4.9)	Home-care package with a weight scale, a blood pressure meter, a mobile phone, and self-care instructions; measurements with the assessment of symptoms 1 time/week; automatic feedback if parameter was within personal targets set by the nurse; the nurse checked the data 1 time/week or more frequently when measurement was beyond target levels or different from the previous one. Nurse of the cardiac team	Multidisciplinary care approach, guidance and support for self-care, patients’ visits to the clinic and by phone calls	94; 6 months: 1.1%	Self-care	European Heart Failure Self-Care Behavior Scale (Jaarsma 2003)

More than half of the studies (*n* = 16) investigated transitional care programs consisting of interventions started during hospitalisation and continued when the patient was home through telephone follow-ups in eight studies [42–46, 55, 57, 59], home visits and phone calls in three studies [49, 51, 65], digital interventions in two studies [62, 63], digital interventions and phone calls in two studies [47, 61], and home care in one study [54] (Tables 1 and 2). Seven studies evaluated home care interventions integrated with phone call follow-ups [43, 46, 55, 57, 59] and digital interventions [48, 52]. Four studies investigated remote care interventions, of which two studies evaluated phone call interventions [41, 60] and two experimented digital interventions [62, 63].

**Table 2:** Characteristics of interventions and outcomes of studies not included in the meta-analysis

	Intervention components															Outcomes		
	Hospital sessions	Home visits	Structured phone calls follow-up	Motivational interviewing	Reminders for medications	Motivational messages	Digital tool	Telemonitoring	Action plan/goal settingon self-care	Education on self-monitoring	Education on lifestyle (diet, PA etc.)	Education on HF, signs and symptoms	Education on medication adherence	Educational materials	Other information on the intervention	Self- management	Self-care maintenance	Self-care behaviors
*Home care*																		
Hoban et al., 2013		●					●	●		●	●		●	●			+ (NR)	
Clark et al., 2015		●	●											●	Stuifbergen’s health Promotion + social and stress support	0.03	0.06	
Masterson Creber et al., 2016		●	●						●		●	●	●				0.08	
Jiang et al., 2021		●			●	●	●	●	●					●	Pill box with alarms	0.001	0.001	
Shao et al., 2013		●	●						●	●	●							+ (NR)
De Souza et all; 2014		●	●								●	●	●		Self-efficacy (Bandura)			MA
Motta et al., 2013		●	●							●	●	●	●					MA
*Transitional care*																		
Balk et al., 2008					●	●	●	●			●			●	TV channel			± (NR)
Hwang et al., 2022	●		●						●		●	●	●		Teach back			< 0.001
Jaarsma et al., 2000b	●	●	●							●	●	●	●	●				0.11
Moon et al., 2018	●		●						●	●	●	●		●	Stress management			< 0.001
Sadeghi Akbari et al., 2019	●		●						●				●	●	Leventhal self-regulation model			< 0.001
Koberich et al., 2015	●		●							●	●	●	●	●				0.043
Hoover et al., 2017	●	●	●		●					●		●	●		Coleman Care Transitions Intervention + medication reconciliation	0.08	0.47	
Zamanzadeh et al., 2013	●		●						●		●	●	●	●	Orem’s self-care theory	< 0.001	< 0.001	
Yu et al., 2015	●	●	●						●	●	●	●			Providing community support services	> 0.05	< 0.05	
Cossette et al., 2016	●		●									●			Self-Determination Theory		MA	
Chen et al., 2018	●		●	●					●		●	●	●			MA	MA	
Leavitt et al., 2020	●	●							●		●	●	●		Home environment examination	MA	MA	
Davis et al., 2012			●						●	●		●	●	●	Cognitive training + environmental manipulation + teach-back	MA	MA	
Evangelista et al., 2015			●				●				●	●				MA	MA	
Wonggom et al., 2020	●						●		●		●	●				MA	MA	
Sun et al., 2019	●		●				●		●		●	●	●			MA	MA	
*Remote care*																		
Brandon et al., 2009			●								●	●	●					0.001
Shearer et al., 2007			●						●	●		●			Rogers’ Science of Unitary Human Beings process			+ (NR)
Vuorinen et al., 2014							●	●		●								0.298
Ware et al., 2022							●	●		●					Automatic self-care instructions	0.40	0.82	

Legend: NR, Not reported; MA, metanalysis

All interventions were delivered by nurses, and, in all studies, nurses provided clinical assessments and educational advice on HF and HF-related self-care, mostly on lifestyle, sign and symptom monitoring and management, and medication adherence (Table 2). The main methods and instruments used for educational purposes were booklets, motivational interviewing, counselling, and goal setting. Digital components varied across the studies: remote monitoring system platform [47, 48, 52, 61–63], mobile app [52, 61, 63], videoconferencing [47], WeChat [61], avatar app on a tablet [64], and TV channels with medication reminders and motivational messages [40]. The theory or framework of reference was reported in six studies (Table 2), including Stuifbergen’s Health Promotion [43], Self-efficacy theory [46], Self-determination theory [44], Rogers’ Science of Unitary Human Beings process [60], and Leventhal self-regulation model [58], and Orem’s self-care theory [66]. These models share the fundamental principle of promoting psychological factors to manage the complexity of chronic diseases and facilitate behaviour change, and some models also emphasize the role of social and environmental support.

The longest follow-up period in each study was one month in four studies [44, 45, 49, 54], five weeks in one study [56], two months in two studies [42, 58], three months in nine studies [41, 47, 48, 53, 55, 59, 60, 64, 66], six months in eight studies [43, 46, 50, 52, 57, 61–63], nine months in two studies [51, 65], and non-defined a priori follow-ups [40] (Table 1).

### Risk of bias

**Fig. 2:**
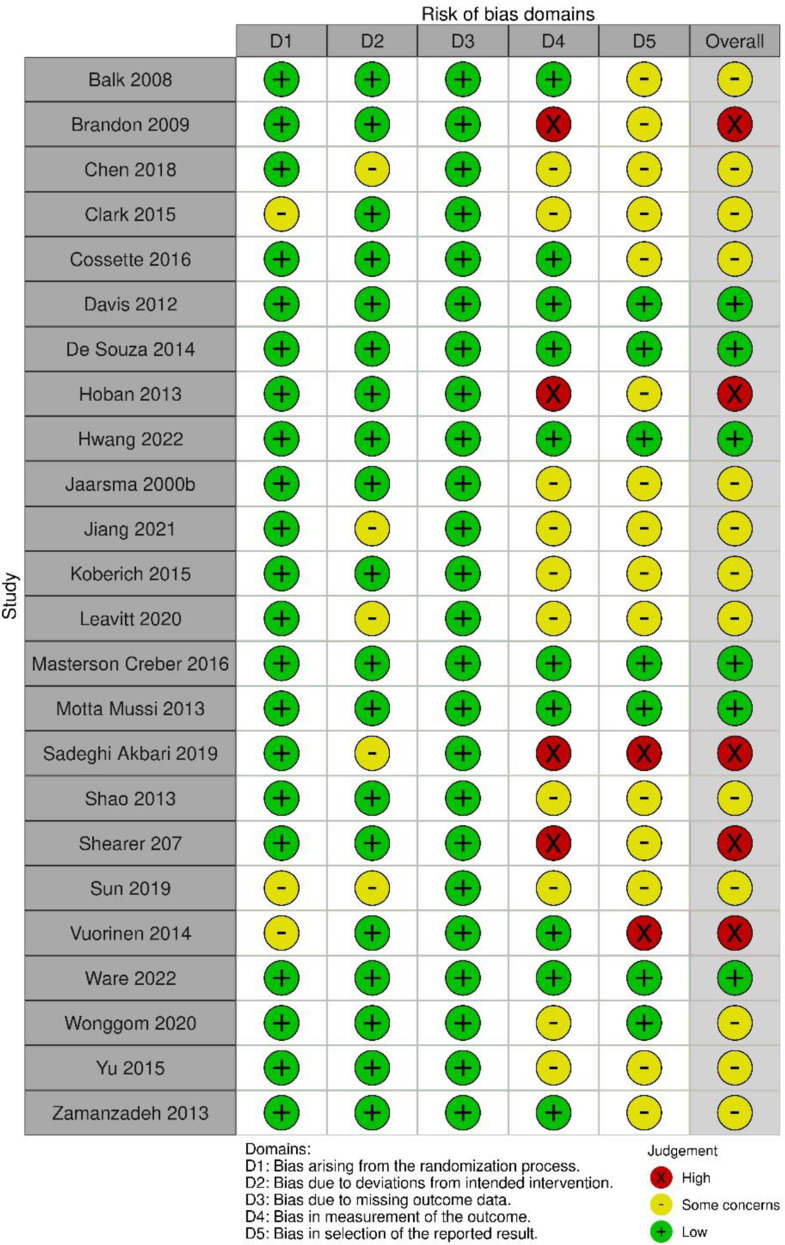
The risk of bias of randomized control studies (RoB 2)

**Fig. 3:**
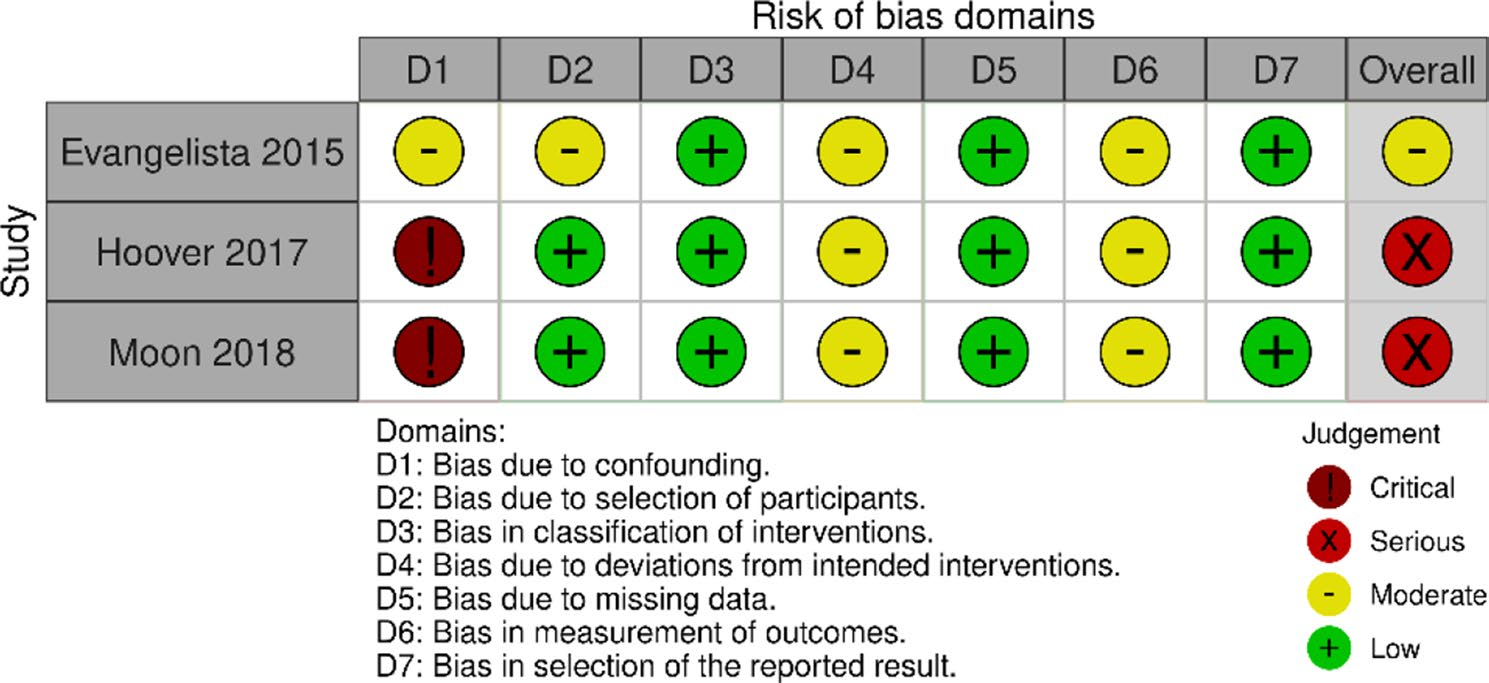
The Risk of Bias in Non-randomized Studies of Interventions (ROBINS-I)

Of the 24 RCTs, we judged five studies to be at high risk of bias. In these studies, a high risk was detected in the domains of “measurement of outcome” in four studies, and of “selection of the reported results” in two studies (Fig. 2). A total of 14 studies were given a moderate risk of bias and six a low risk of bias. Out of three quasi-experimental studies, one study was evaluated at moderate risk of bias, and two were evaluated as being at serious risk of bias (Fig. 3). No information regarding controlling confounding factors or post-hoc analysis for those factors that confound the intervention effects (that is, factors that can affect self-care or intervention effects) was presented in the studies.

### Effects of interventions

The most commonly used instruments for measuring self-care were the Self-Care of Heart Failure Index (SCHFI) [33] utilized in its original or modified versions in 15 studies [42–45, 47–49, 52, 54, 55, 61, 63–66] and the European Heart Failure Self Care Behaviour questionnaire (EHFScBs) [51] employed in its original or modified version in nine studies [40, 46, 50, 53, 56–59, 62]. Validated versions were available in Chinese, French, and Farsi for the SCHFI, and in Korean, German, and Portuguese for the EHFScBs.

### Self-care maintenance

**Fig. 4:**
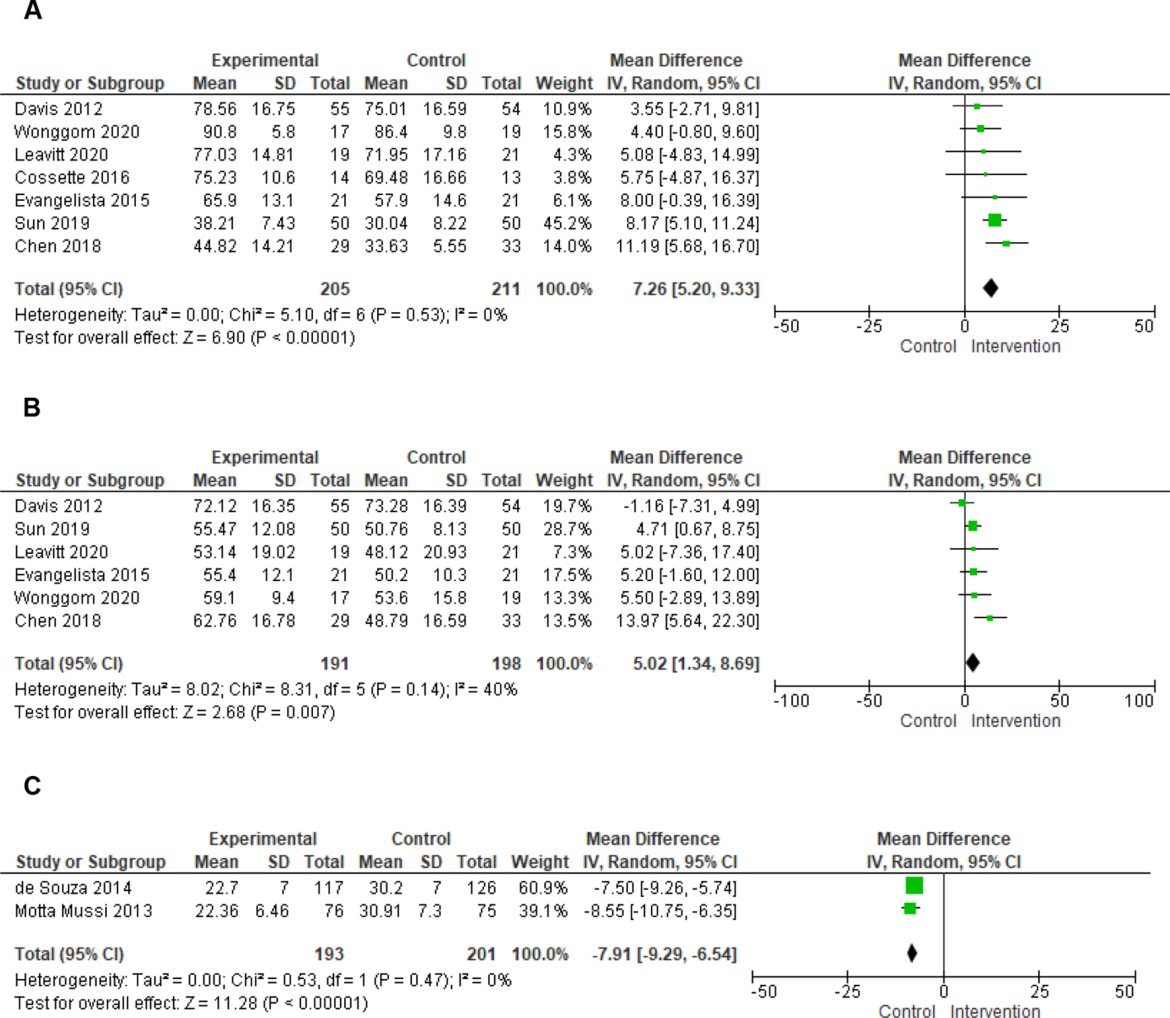
Forest plots. **(A)** Self-care maintenance – transitional care. **(B)** Self-care management – transitional care. **FC.** Self-care behaviours – home care interventions

Fifteen studies reported self-care maintenance measured with SCHFI [42–45, 47–49, 52, 54, 55, 61, 63–66], of which 10 investigated transitional care [42, 44, 45, 47, 49, 54, 61, 64–66], four home care [43, 48, 52, 55], and one a digital interventions [63]. Regarding transitional care, seven of 10 studies were included in the meta-analyses [42, 44, 45, 47, 54, 61, 64], using SCHFI version 6.2 with a scoring range of 10–40 and a clinically relevant improvement of 8 standardized points on a range of 0 to 100 [33]. Transitional care with at-home after discharge care may result in little to no difference in self-care maintenance when compared to usual care (Fig. 4, Forest Plot A: MD 7.26, 95% CI 5.20, 9.33, I^2^ = 0%, Table 3). Sensitivity analyses confirmed the robustness of the results, by removing the quasi-experimental study [47] (MD: 7.20, 95% CI 5.04, 9.36, I^2^ = 1%, *p* <.00001), while no high-risk studies were included in the meta-analysis. Of the remaining three studies not included in the meta-analysis, two reported statistically significant results in favour of transitional care (*p* <.001 [66], *p* <.05 [65]) and one study showed no difference between the intervention and control group (*p* =.47) [49] (Table 2).

**Table 3:** GRADE evidence profile

Certainty assessment							№ of patients		Effect	Certainty
№ of studies	Study design	Risk of bias	Inconsistency	Indirectness	Imprecision	Other considerations	Nursing intervertions delivered totally or partially at home	usual care	Absolute (95% CI)	
**Self-care Maintenance**										
7	randomised trials	serious^a^	not serious	not serious	serious^b^	none	205	211	MD **7.26 higher** (5.2 higher to 9.33 higher)	⨁⨁◯◯ Low
**Self-care Management**										
6	randomised trials	serious^a^	not serious	not serious	serious^c^	none	194	198	MD **5.02 higher** (1.34 higher to 8.69 higher)	⨁⨁◯◯ Low
**Self-care behaviours**										
2	randomised trials	not serious	not serious	serious^d^	not serious	none	193	201	MD **7.91 lower** (9.29 lower to 6.54 lower)	⨁⨁⨁◯ Moderate

**CI**: confidence interval; **MD**: mean difference

**Explanations**

a. Most studies were at moderate risk of bias

b. Choosing a Δ of 3.2 (derived from a clinically relevant improvement of 8 standardized points suggested by the authors of the instrument) and using the standard deviations associated with the studies included in the analysis, the optimal information size (OIS) criterion is not met

c. Choosing a Δ of 1.6 (derived from a clinically relevant improvement of 8 standardized points suggested by the authors of the instrument) and using the standard deviations associated with the studies included in the analysis, the optimal information size (OIS) criterion is not met

d. The two studies included only patients with an LVEF lower than 45% and hospitalized for acute decompensation. These criteria do not fully represent the overall population with HF; therefore, considering the applicability of these results, we deemed to downgrade indirectness by one level

Home care interventions were reported effective in improving self-care maintenance compared to usual care in association with digital interventions (telemonitoring equipment, smartphone app) in two studies (p or CI95%=not reported [48], *p* =.001 [52]), while not effective in association with phone calls in other two studies (*p* =.06 [43], *p* =.08 [55]). The study with remote care that investigated the digital intervention alone showed no difference between the intervention and control group (*p* =.82 [63]), (Table 2).

### Self-care management

Thirteen studies reported self-care management measured with SCHFI [42, 43, 45, 47–49, 52, 54, 61, 63–66], of nine which investigated transitional care [42, 45, 47, 49, 54, 61, 64–66], three home care interventions [43, 48, 52], and one a remote care intervention [63]. Among studies on transitional care, the pooled effect size was calculated for six studies [42, 45, 47, 54, 61, 64], using SCHFI version 6.2 with a scoring range of 5–20 and a clinically relevant improvement of 8 standardized points on a range of 0 to 100 [33]. The meta-analysis revealed thattransitional care with at-home after discharge care may result in little to no difference in self-care management (Fig. 4, Forest Plot B: MD 5.02, 95% CI 1.34, 8.69, I^2^ = 40%, Table 3). All sensitivity analyses confirmed the robustness of results, by removing the quasi-experimental study [47] (MD: 5.11, 95% CI 0.51, 9.71, I^2^ = 52%, *p* =.03), while no high-risk studies were included in the meta-analysis. Of the three studies not included in the meta-analysis, one showed results in favour of transitional care (*p* <.001 [66]), and two showed no difference between the intervention and control group (*p* >.05 [65], *p* =.08 [49]). The three studies investigating home care combined with digital interventions [48, 52] or phone calls [43] reported statistically significant results in favour of interventions (p or CI95%=not reported [48], *p* =.001 [52], *p* =.03 [43]), while the study that investigated the digital intervention alone showed no difference between groups (*p* =.40 [63]), (Table 2).

### Self-care behaviours

Twelve studies reported data on self-care behaviours measured with instruments different from SCHFI, and specifically in nine studies [40, 46, 50, 53, 56–59, 62] with the European Heart Failure Self Care Behaviour questionnaire (EHFScBs), while in others with the “Heart Failure Self-Care Behaviour Scale” [51], “Self-care Behaviours Scale” [41], and “Self-Management of Heart Failure scale” [60]. Six studies evaluated transitional care with at-home aftercare. Of these, five studies used original or modified versions of the EHFScBs, of which four reported statistically significant results in favour of hospital educational sessions with post-discharge phone calls (p.<0.001 [50, 53, 56], *p* =.04 [58]), and one reported no difference when evaluating hospital sessions combined with a digital intervention after discharge (TV channel, remote monitoring etc., p or CI95%=not reported, Table 2) [40]. One study using the “Heart Failure Self-Care Behaviour Scale” reported no improvement in self-care as a result of transitional care with post-discharge home visits and phone calls (*p* =.11 [51]).

Three studies investigated home care combined with home visits and phone calls measuring self-care with EHFScBs. The meta-analysis of the two studies adopting the same EHFScBs 12-item version with a reverse scoring range of 12–60 (higher scores, lower self-care) and a clinically relevant reduction of 5.75 standardized points on a range of 0 to 100 [46, 57, 67], revealed that home care consisting of home visits and phone calls likely increase self-care (Fig. 4, Forest Plot C: MD -7.91, 95% CI -9.29, -6.54; I^2^ = 0%; Additional File 4, Table 3). This result is further confirmed by the third study using a different version of EHFScBs not included in the metanalysis (p or CI95%=not reported [59]). Among the three studies on remote care interventions, two evaluated phone call programs using the “Self-care Behaviours Scale” [41] and “Self-Management of Heart Failure scale” [60] showed an improvement of self-care in favour of the intervention (*p* =.001 [41], p or CI95%=not reported [60]), while one study on remote monitoring [62] showed no differences (*p* =.298) between the intervention and control group in self-care measured with EHFScBs (Table 2).

## Discussion

This review systematically summarised the effect of nursing interventions on self-care of patients with HF, that allowed patients to receive care by staying at home during out-of-hospital care. The results of the meta-analyses showed that transitional care aimed at caring for patients at their home after discharge through phone calls, digital interventions, home visits or a combination thereof, may result in little to no difference in self-care maintenance (MD 7.26, 95% CI 5.20, 9.33) and self-care management (MD 5.02, 95% CI 1.34, 8.69) in patients with HF, indicating a small and non-clinically relevant effect. The results of four of six studies assessing self-care behaviours with other self-care instruments showed a positive effect in favour of transitional care combined with post-discharge phone calls, even though two were at moderate risk of bias and one was at high risk of bias.

Regarding home care interventions consisting of home visits combined with phone calls or digital interventions, two studies reported consistent results on the effectiveness in improving self-care management, even though at moderate risk of bias. Regarding self-care behaviours measured with instruments different from SCHIFI, a meta-analysis of two studies showed that they are likely increased by a structured program consisting of home visits and phone calls. Differently, contrasting results emerged on self-care maintenance.

As for remote care interventions, phone calls alone were effective in improving self-care behaviours measured with instruments different from SCHIFI, however, both studies were at high risk of bias, thus potentially misleading the results. Digital interventions were not effective on all outcomes, as reported by two studies, one at moderate and one at high risk of bias.

This suggests that patients with HF could slightly improve their self-care while being cared for at home through different combinations of nursing interventions, mostly delivered as transitional care, home-based interventions, and phone calls, and therefore learning and improving their abilities to maintain clinical stability, monitor signs and symptoms, and interpret signs and symptoms to identify the best management plan. However, caution is needed since the low or moderate quality of evidence and moderate or high risk of bias.

Previous reviews about the efficacy of nursing intervention in improving self-care in patients with HF supported our results, even though they are not completely comparable with our review, as they also included interventions totally delivered at the hospital and in outpatient setting [21, 68, 69], or self-care results measured only through SCHIFI [21]. Specifically, a previous meta-analysis demonstrated the efficacy of nurse-led self-care interventions in improving self-care [21], as did a review of nurse-led transitional care [70]. However, another recent review found no improvement in the self-care of patients with advanced HF when they are managed with a nurse case management program in primary care [68], even though these results are of questionable significance, as they are derived from three studies with a heterogeneity of 97% [68].

Despite the beneficial results, due care should be paid in applying these interventions to clinical practice for several reasons. First, there is a low certainty of evidence, meaning that the true effect may differ from the estimated effect, except for home-based interventions in self-care behaviours. Second, narratively summarized studies revealed variations in outcomes related to self-care maintenance and self-care management for transitional care and home-based interventions and to self-care behaviours for transitional care and remote interventions.

As for other implications for practice and research, we found that digital interventions alone, mainly as remote monitoring, are not effective in improving self-care. Differently, they tend to be effective when they are included in transitional care programs or combined with home visits. This calls for the need for further research on how to make effective digital interventions when delivered completely in a remote way with no face-to-face visits, also considering previous contrasting results. Indeed, recent meta-analyses demonstrated that self-management through digital interventions, as well as nurse-led telecoaching [71] can reduce mortality and hospital readmission and improve medication adherence and self-care behaviours [69].

In prior studies, patient recruitment was predominantly hospital-based, which could be considered either a pro or a con. The recruitment during hospitalization could be effective in targeting patients at high risk of readmission. Furthermore, patients are more sensitive to acquiring knowledge about self-care, as the acute event makes self-care salient to patients. This is in contrast to those in a clinical stability situation who may be less aware of self-care relevance [72]. However, hospital-based recruitment represents a reactive strategy to address the issue of hospitalization rates, as it does not enable the identification of patients with an adequate time frame to enhance their self-care abilities before experiencing an acute event. Therefore, hospital-based recruitment could be complemented with proactive strategies to prevent hospitalizations. Indeed, patients with HF may also benefit from community-based recruitment through general practitioners and primary care health care professionals. In fact, in this manner, patients can be identified before the acute event, thus gaining time to educate them on self-care in order to maintain clinical stability, prevent complications, and increase the likelihood of avoiding hospitalization due to decompensation [73]. Relatedly, most interventions investigated transitional care programs, while very few interventions took place involving nurses working in primary care, home care, and general practice. The involvement of primary care nurses and community nurses might help foster at-home interventions and create networks with specialised care to ensure continuity of care and proper support for the long-term development of self-care abilities beyond the first few months after hospitalisation [27].

This review provides different effective solutions for policymakers and stakeholders to integrate the role of nurses into interventions to address patients’ educational needs on self-care at patient’s home. Nursing interventions aimed at caring for patients at their home could especially help older, frail and isolated adults with HF, living in remote areas that struggle to reach services. Because the number of older people with disabilities and without social support is increasing, and it is fundamental to strengthen approaches and interventions that can facilitate reaching those facing accessibility barriers or residing in remove areas.

Despite variations in components and timing of interventions among the studies included (i.e. transitional care, home visits, remote care), we found consistent characteristics that encompass all the interventions. These common features could help explain why at-home interventions were effective on self-care. For example, the continuous relationship with nurses ensured by a structured follow-up, mostly consisting of home visits and phone calls, could have helped patients to feel supported and informed, as well as to stay engaged in their health pathway [72, 74]. Indeed, this relationship could have been limited in digital interventions that resulted in no improvement in self-care when not combined with transitional care and home visits. In addition, almost all the interventions included an educational component aimed at fostering sustained behavioural change. Specifically, the contents covered were mostly on signs and symptoms, medication adherence, and lifestyles, including a healthy diet and low-salt diet, physical activity, stop smoking. The education was delivered through methods like motivational interviewing, action plans, goal setting, and decisional algorithms. This could have supported the patient to find and leverage their resources to foster or acquire new self-care abilities [22]. The incorporation of educational components, coupled with structured follow-up, may elucidate the long-term efficacy of the intervention [72]. This approach ensures that patients are not left to navigate in the extended learning process of self-care behaviours.

This review has some limitations. Three quasi-experimental studies were included that might not ensure the proper methods to draw accurate conclusions. However, in the meta-analysis, sensitivity analyses were performed excluding these studies, and the robustness of the results was confirmed. Furthermore, the best type of interventions could not be identified due to the large variety of models, and therefore we were not able to find the most effective interventions. However, we were able to isolate digital interventions as those less effective in improving self-care. In the studies under review, the level of self-care that participants reported might be influenced by the Hawthorne effect. This is because participants, being aware of their participation in the study when responding to the self-care questionnaire, might introduce recall bias to their reported self-care behaviours.

## Conclusion

Nursing interventions performed as transitional care integrated with phone calls, home visits, and digital interventions, home visits with phone calls and digital interventions, and phone call programs could slightly improve self-care in patients with HF. However, caution should be paid since the certainty of the evidence was rated low for self-care management and self-care maintenance and studies not included in the meta-analysis for all outcomes were mostly at moderate risk of bias and few at high risk of bias. Despite the differences in the interventions, a supportive relationship and structured follow-up with the nurse can contribute to improving the patient´s self-care. Studies should focus on investigating how to make effective digital interventions alone and the efficacy on different dimensions of self-care. Further studies are also needed to examine the relationship between adequate self-care at baseline and other outcomes, such as hospitalization rates and quality of life to know whether those with adequate self-care need to be excluded from interventions.

## Electronic supplementary material

Below is the link to the electronic supplementary material.

Supplementary Material 1

## Data Availability

The data supporting the findings of this study are available within the article and its Additional material. The datasets generated during and/or analysed during the current review are available from the first author on reasonable request.
