# Supplementary material for: The effectiveness of nursing interventions to improve self-care for patients with heart failure at home: a systematic review and meta-analysis
Source: BMC Nurs. 2025 Mar 14;24:286. doi: 10.1186/s12912-025-02867-7 (PMC11908091; doi:10.1186/s12912-025-02867-7)
Supplement: Supplementary file 1 — Supplementary Material 1 [file 12912_2025_2867_MOESM1_ESM.docx]

**Additional Files**

**Index**

[Additional file 1. The Preferred Reporting Items for Systematic Reviews and Meta-Analyses checklist 2](#_Toc171418507)

[Additional file 2. Search strings 4](#_Toc171418508)

[Additional file 3. List of excluded studies from the full-text screening and the main reason of exclusion 11](#_Toc171418509)

# Additional file 1. The Preferred Reporting Items for Systematic Reviews and Meta-Analyses checklist

| **Section and Topic** | **Item #** | **Checklist item** | **Location where item is reported (Page(s))** |
| --- | --- | --- | --- |
| **TITLE** | | |  |
| Title | 1 | Identify the report as a systematic review. | 1 |
| **ABSTRACT** | | |  |
| Abstract | 2 | See the PRISMA 2020 for Abstracts checklist. | 2-3 |
| **INTRODUCTION** | | |  |
| Rationale | 3 | Describe the rationale for the review in the context of existing knowledge. | 4-6 |
| Objectives | 4 | Provide an explicit statement of the objective(s) or question(s) the review addresses. | 4-6 |
| **METHODS** | | |  |
| Eligibility criteria | 5 | Specify the inclusion and exclusion criteria for the review and how studies were grouped for the syntheses. | 7 |
| Information sources | 6 | Specify all databases, registers, websites, organisations, reference lists and other sources searched or consulted to identify studies. Specify the date when each source was last searched or consulted. | 7-8 Additional file 2 |
| Search strategy | 7 | Present the full search strategies for all databases, registers and websites, including any filters and limits used. |  |
| Selection process | 8 | Specify the methods used to decide whether a study met the inclusion criteria of the review, including how many reviewers screened each record and each report retrieved, whether they worked independently, and if applicable, details of automation tools used in the process. | 7-8 Additional file 2 |
| Data collection process | 9 | Specify the methods used to collect data from reports, including how many reviewers collected data from each report, whether they worked independently, any processes for obtaining or confirming data from study investigators, and if applicable, details of automation tools used in the process. | 8 |
| Data items | 10a | List and define all outcomes for which data were sought. Specify whether all results that were compatible with each outcome domain in each study were sought (e.g., for all measures, time points, analyses), and if not, the methods used to decide which results to collect. | 8 |
|  | 10b | List and define all other variables for which data were sought (e.g., participant and intervention characteristics, funding sources). Describe any assumptions made about any missing or unclear information. | 8 |
| Study risk of bias assessment | 11 | Specify the methods used to assess risk of bias in the included studies, including details of the tool(s) used, how many reviewers assessed each study and whether they worked independently, and if applicable, details of automation tools used in the process. | 8 |
| Effect measures | 12 | Specify for each outcome the effect measure(s) (e.g., risk ratio, mean difference) used in the synthesis or presentation of results. | 8-9 |
| Synthesis methods | 13a | Describe the processes used to decide which studies were eligible for each synthesis (e.g., tabulating the study intervention characteristics and comparing against the planned groups for each synthesis (item #5)). | 9-10 |
|  | 13b | Describe any methods required to prepare the data for presentation or synthesis, such as handling of missing summary statistics, or data conversions. | 9-10 |
|  | 13c | Describe any methods used to tabulate or visually display results of individual studies and syntheses. | 9-10 |
|  | 13d | Describe any methods used to synthesize results and provide a rationale for the choice(s). If meta-analysis was performed, describe the model(s), method(s) to identify the presence and extent of statistical heterogeneity, and software package(s) used. | 9-10 |
|  | 13e | Describe any methods used to explore possible causes of heterogeneity among study results (e.g. subgroup analysis, meta-regression). | NA |
|  | 13f | Describe any sensitivity analyses conducted to assess robustness of the synthesized results. | 9-10 |
| Reporting bias assessment | 14 | Describe any methods used to assess risk of bias due to missing results in a synthesis (arising from reporting biases). | 8 |
| Certainty assessment | 15 | Describe any methods used to assess certainty (or confidence) in the body of evidence for an outcome. | 8 |
| **RESULTS** | | |  |
| Study selection | 16a | Describe the results of the search and selection process, from the number of records identified in the search to the number of studies included in the review, ideally using a flow diagram. | 10, Figure 1 |
|  | 16b | Cite studies that might appear to meet the inclusion criteria, but which were excluded, and explain why they were excluded. | Additional file 3 |
| Study characteristics | 17 | Cite each included study and present its characteristics. | 10 – Table 1 |
| Risk of bias in studies | 18 | Present assessments of risk of bias for each included study. | 11, Figure 2 and 3 |
| Results of individual studies | 19 | For all outcomes, present, for each study: (a) summary statistics for each group (where appropriate) and (b) an effect estimate and its precision (e.g. confidence/credible interval), ideally using structured tables or plots. | 11-14, Table 2 Figure 4 |
| Results of syntheses | 20a | For each synthesis, briefly summarise the characteristics and risk of bias among contributing studies. | 11-14, Table 2 Figure 4 |
|  | 20b | Present results of all statistical syntheses conducted. If meta-analysis was done, present for each the summary estimate and its precision (e.g., confidence/credible interval) and measures of statistical heterogeneity. If comparing groups, describe the direction of the effect. | 11-14, Table 2 Figure 4 |
|  | 20c | Present results of all investigations of possible causes of heterogeneity among study results. | NA |
|  | 20d | Present results of all sensitivity analyses conducted to assess the robustness of the synthesized results. | 11-14 |
| Reporting biases | 21 | Present assessments of risk of bias due to missing results (arising from reporting biases) for each synthesis assessed. | 11, Figure 2 and 3 |
| Certainty of evidence | 11-14 | Present assessments of certainty (or confidence) in the body of evidence for each outcome assessed. | 11-14, Table 3 |
| **DISCUSSION** | | |  |
| Discussion | 23a | Provide a general interpretation of the results in the context of other evidence. | 14-18 |
|  | 23b | Discuss any limitations of the evidence included in the review. | 17-18 |
|  | 23c | Discuss any limitations of the review processes used. | 17-18 |
|  | 23d | Discuss implications of the results for practice, policy, and future research. | 14-18 |
| **OTHER INFORMATION** | | |  |
| Registration and protocol | 24a | Provide registration information for the review, including register name and registration number, or state that the review was not registered. | 7 |
|  | 24b | Indicate where the review protocol can be accessed, or state that a protocol was not prepared. | 7 |
|  | 24c | Describe and explain any amendments to information provided at registration or in the protocol. | No amendments except for title |
| Support | 25 | Describe sources of financial or non-financial support for the review, and the role of the funders or sponsors in the review. | 19 |
| Competing interests | 26 | Declare any competing interests of review authors. | 19 |
| Availability of data, code and other materials | 27 | Report which of the following are publicly available and where they can be found: template data collection forms; data extracted from included studies; data used for all analyses; analytic code; any other materials used in the review. | Information retrieved from published articles included |

# Additional file 2. Search strings

Databases:

1. Medline (Ovid)
2. Cochrane Library (Wiley)
3. Web of Science Core Collection (Clarivate Analytics)
4. PsycInfo (Ebsco)
5. Cinahl (Ebsco)

Total number of hits:

- Before deduplication: 5,401
- After deduplication: 3,104

Comments:

Deduplication based on the method described in:
Bramer, W. M., Giustini, D., de Jonge, G. B., Holland, L., & Bekhuis, T. (2016). De-duplication of database search results for systematic reviews in EndNote. *Journal of the Medical Library Association: JMLA*, 104(3), 240–243. doi:10.3163/1536-5050.104.3.014

One final, extra step was added to compare DOIs.

**1. Medline**

| Database(s): **Ovid MEDLINE(R) and Epub Ahead of Print, In-Process, In-Data-Review & Other Non-Indexed Citations and Daily**from 1946  Search Strategy:   \| **#** \| **Searches** \| \| --- \| --- \| \| 1 \| exp Heart Failure/ \| \| 2 \| ((cardiac or heart or myocardi* or ventric*) adj3 (decompensat* or dysfunction* or insufficien* or failure)).ti,ab,kf. \| \| 3 \| 1 or 2 \| \| 4 \| exp Self Care/ or Self Management/ or Self-Examination/ \| \| 5 \| (selfadministrat* or self-administrat* or selfcare or self-care or self-caring or self-examination or selfexamination or selfhelp* or self-help or selfmanag* or self-manag* or self-monitor* or selftreat* or self-treat*).ti,ab,kf. \| \| 6 \| ((home* or symptom* or side effect*) adj2 (manag* or monitor* or telemonitor*)).ti,ab,kf. \| \| 7 \| Telemedicine/ or Telenursing/ or Telerehabilitation/ or exp Remote Consultation/ \| \| 8 \| (distance counsel?ing or distance consultation* or e consultation* or econsultation* or e counsel?ing or ecounsel?ing or e health* or ehealth* or e therapies or e therapy or etherap* or e visit* or evisit* or m health or mhealth or mobile counsel?ing or mobile consultation* or remote consultation* or remote counsel?ing or telecare or tele care or tele health* or telehealth* or tele consultation* or teleconsultation* or tele medicine or telemedicine or tele nurs* or telenurs* or tele rehabilitation or telerehabilitation).ti,ab,kf. \| \| 9 \| Health Knowledge, Attitudes, Practice/ or Health Behavior/ \| \| 10 \| (health* adj3 (attitud* or behavio?r* or knowledge or practice*)).ti,ab,kf. \| \| 11 \| Patient Compliance/ or Medication Adherence/ or "Treatment Adherence and Compliance"/ \| \| 12 \| ((treatment* or regimen or medication* or patient*) adj3 (complian* or adher* or noncomplian* or nonadher*)).ti,ab,kf. \| \| 13 \| Exercise/ \| \| 14 \| (exercis* or (physical* adj2 (activ* or inactiv* or training))).ti,ab,kf. \| \| 15 \| Healthy Lifestyle/ or Life Style/ \| \| 16 \| (lifestyle* or life-style*).ti,ab,kf. \| \| 17 \| Drinking Behavior/ or Alcohol Drinking/ or Alcohol Abstinence/ or Temperance/ or Binge Drinking/ \| \| 18 \| (drink* adj2 (behavio?r* or binge* or excessive* or harm* or hazard* or heavy or high risk* or problem*)).ti,ab,kf. \| \| 19 \| ((alcohol* or binge*) adj3 (addict* or abus* or abstinen* or consumption* or dependen* or drink* or intake* or misus* or problem* or rehabilit* or restrict*)).ti,ab,kf. \| \| 20 \| (alcohol* adj1 (use* or using)).ti,ab,kf. \| \| 21 \| temperance*.ti,ab,kf. \| \| 22 \| exp Diet/ or Feeding Behavior/ or Weight Reduction Programs/ or exp Body Weight/ \| \| 23 \| ((health* or unhealth*) adj2 (diet* or eat* or food* or habit*)).ti,ab,kf. \| \| 24 \| ((eat* or diet* or food? or feed*) adj3 (behavio?r* or habit* or pattern*)).ti,ab,kf. \| \| 25 \| ((food? or diet*) adj3 (choice* or frequenc* or intake* or intervention* or modification* or therap*)).ti,ab,kf. \| \| 26 \| ((calori* or fruit* or fat* or fiber* or fibre* or portion* or salt or sodium or sugar* or vegetable*) adj3 (choice* or consum* or decreas* or diet or discourage* or eat* or frequenc* or intake or increase* or limit* or less or lessen or number* or portion* or preference* or reduc* or restriction* or serving* or size*)).ti,ab,kf. \| \| 27 \| ((daily adj1 weigh*) or (weight adj2 (monitor* or loss or reduct*))).ti,ab,kf. \| \| 28 \| Smoking Cessation/ or Smoking Reduction/ or "Tobacco Use Cessation"/ or "Tobacco Use"/ or Smoking/ \| \| 29 \| (anti-smoking or antismoking or cigar* or cigarette* or nicotine or smoking or smoker? or snuff or snus or tobacco).ti,ab,kf. \| \| 30 \| exp Treatment Refusal/ \| \| 31 \| (annual* adj3 (vaccination* or immunization* or immunisation* or shot?)).ti,ab,kf. \| \| 32 \| or/4-31 \| \| 33 \| Practice Patterns, Nurses'/ or Nurse's Role/ or exp Nurses/ or exp Nursing/ \| \| 34 \| nursing.fs. \| \| 35 \| (nurse* or nursing or telenurs*).ti,ab,kf. \| \| 36 \| or/33-35 \| \| 37 \| 3 and 32 and 36 \| |
| --- | --- | --- | --- | --- | --- | --- | --- | --- | --- | --- | --- | --- | --- | --- | --- | --- | --- | --- | --- | --- | --- | --- | --- | --- | --- | --- | --- | --- | --- | --- | --- | --- | --- | --- | --- | --- | --- | --- | --- | --- | --- | --- | --- | --- | --- | --- | --- | --- | --- | --- | --- | --- | --- | --- | --- | --- | --- | --- | --- | --- | --- | --- | --- | --- | --- | --- | --- | --- | --- | --- | --- | --- | --- | --- | --- | --- |

**2. Cochrane Library**

| \| ID \| Search \| \| --- \| --- \| \| #1 \| ((cardiac or heart or myocardi* or ventric*) NEAR/3 (decompensat* or dysfunction* or insufficien* or failure)):ti,ab,kw \| \| #2 \| (selfadministrat* or self-administrat* or selfcare or self-care or self-caring or self-examination or selfexamination or selfhelp* or self-help or selfmanag* or self-manag* or self-monitor* or selftreat* or self-treat*):ti,ab,kw \| \| #3 \| ((home* or symptom* or "side effect" or "side effects") NEAR/2 (manag* or monitor* or telemonitor*)):ti,ab,kw \| \| #4 \| ("distance counsel?ing" or (distance NEXT consultation*) or (e NEXT consultation*) or econsultation* or "e counsel?ing" or ecounsel?ing or (e NEXT health*) or ehealth* or "e therapies" or "e therapy" or etherap* or (e NEXT visit*) or evisit* or "m health" or mhealth or "mobile counsel?ing" or (mobile NEXT consultation*) or (remote NEXT consultation*) or "remote counsel?ing" or telecare or "tele care" or (tele NEXT health*) or telehealth* or (tele NEXT consultation*) or teleconsultation* or "tele medicine" or telemedicine or (tele NEXT nurs*) or telenurs* or "tele rehabilitation" or telerehabilitation):ti,ab,kw \| \| #5 \| (health* NEAR/3 (attitud* or behavio?r* or knowledge or practice*)):ti,ab,kw \| \| #6 \| ((treatment* or regimen or medication* or patient*) NEAR/3 (complian* or adher* or noncomplian* or nonadher*)):ti,ab,kw \| \| #7 \| (exercis* or (physical* NEAR/2 (activ* or inactiv* or training))):ti,ab,kw \| \| #8 \| (lifestyle* or life-style*):ti,ab,kw \| \| #9 \| (drink* NEAR/2 (behavio?r* or binge* or excessive* or harm* or hazard* or heavy or (high NEXT risk*) or problem*)):ti,ab,kw \| \| #10 \| ((alcohol* or binge*) NEAR/3 (addict* or abus* or abstinen* or consumption* or dependen* or drink* or intake* or misus* or problem* or rehabilit* or restrict*)):ti,ab,kw \| \| #11 \| (alcohol* NEAR/1 (use* or using)):ti,ab,kw \| \| #12 \| temperance*:ti,ab,kw \| \| #13 \| ((health* or unhealth*) NEAR/2 (diet* or eat* or food* or habit*)):ti,ab,kw \| \| #14 \| ((eat* or diet* or food? or feed*) NEAR/3 (behavio?r* or habit* or pattern*)):ti,ab,kw \| \| #15 \| ((food? or diet*) NEAR/3 (choice* or frequenc* or intake* or intervention* or modification* or therap*)):ti,ab,kw \| \| #16 \| ((calori* or fruit* or fat* or fiber* or fibre* or portion* or salt or sodium or sugar* or vegetable*) NEAR/3 (choice* or consum* or decreas* or diet or discourage* or eat* or frequenc* or intake or increase* or limit* or less or lessen or number* or portion* or preference* or reduc* or restriction* or serving* or size*)):ti,ab,kw \| \| #17 \| ((daily NEAR/1 weigh*) or (weight NEAR/2 (monitor* or loss or reduct*))):ti,ab,kw \| \| #18 \| (anti-smoking or antismoking or cigar* or cigarette* or nicotine or smoking or smoker? or snuff or snus or tobacco):ti,ab,kw \| \| #19 \| (annual* NEAR/3 (vaccination* or immunization* or immunisation* or shot?)):ti,ab,kw \| \| #20 \| #2 OR #3 OR #4 OR #5 OR #6 OR #7 OR #8 OR #9 OR #10 OR #11 OR #12 OR #13 OR #14 OR #15 OR #16 OR #17 OR #18 OR #19 \| \| #21 \| (nurse* or nursing or telenurs*):ti,ab,kw \| \| #22 \| #1 AND #20 AND #21 \| \| #23 \| (clinicaltrials or trialsearch):so \| \| #24 \| #22 NOT #23 \| |
| --- | --- | --- | --- | --- | --- | --- | --- | --- | --- | --- | --- | --- | --- | --- | --- | --- | --- | --- | --- | --- | --- | --- | --- | --- | --- | --- | --- | --- | --- | --- | --- | --- | --- | --- | --- | --- | --- | --- | --- | --- | --- | --- | --- | --- | --- | --- | --- | --- | --- | --- |

**3. Web of Science Core Collection**

| **22** #19 AND #20 AND #21  1,156  **21** TS=(nurse* OR nursing OR telenurs*)  360,098  **20** TI=((cardiac OR heart OR myocardi* OR ventric*) NEAR/3 (decompensat* OR dysfunction* OR insufficien* OR failure)) OR AB=((cardiac OR heart OR myocardi* OR ventric*) NEAR/3 (decompensat* OR dysfunction* OR insufficien* OR failure)) OR AK=((cardiac OR heart OR myocardi* OR ventric*) NEAR/3 (decompensat* OR dysfunction* OR insufficien* OR failure))  280,839  **19** #18 OR #17 OR #16 OR #15 OR #14 OR #13 OR #12 OR #11 OR #10 OR #9 OR #8 OR #7 OR #6 OR #5 OR #4 OR #3 OR #2 OR #1  2,641,991  **18** TS=(selfadministrat* OR self-administrat* OR selfcare OR self-care OR self-caring OR self-examination OR selfexamination OR selfhelp* OR self-help OR selfmanag* OR self-manag* OR self-monitor* OR selftreat* OR self-treat*)  95,018  **17** TS=(annual* NEAR/3 (vaccination* OR immunization* OR immunisation* OR shot$))  1,638  **16** TS=(anti-smoking OR antismoking OR cigar* OR cigarette* OR nicotine OR smoking OR smoker$ OR snuff OR snus OR tobacco)  454,557  **15** TS=((daily NEAR/1 weigh*) OR (weight NEAR/2 (monitor* OR loss OR reduct*)))  175,965  **14** TS=((calori* OR fruit* OR fat* OR fiber* OR fibre* OR portion* OR salt OR sodium OR sugar* OR vegetable*) NEAR/3 (choice* OR consum* OR decreas* OR diet OR discourage* OR eat* OR frequenc* OR intake OR increase* OR limit* OR less OR lessen OR number* OR portion* OR preference* OR reduc* OR restriction* OR serving* OR size*))  701,717  **13** TS=((food$ OR diet*) NEAR/3 (choice* OR frequenc* OR intake* OR intervention* OR modification* OR therap*))  197,862  **12** TS=((eat* OR diet* OR food$ OR feed*) NEAR/3 (behavio$r* OR habit* OR pattern*))  146,921  **11** TS=((health* OR unhealth*) NEAR/2 (diet* OR eat* OR food* OR habit*))  65,795  **10** TS=temperance*  1,087  **9** TS=(alcohol* NEAR/1 (use* OR using))  74,632  **8** TS=((alcohol* OR binge*) NEAR/3 (addict* OR abus* OR abstinen* OR consumption* OR dependen* OR drink* OR intake* OR misus* OR problem* OR rehabilit* OR restrict*))  143,199  **7** TS=(drink* NEAR/2 (behavio$r* OR binge* OR excessive* OR harm* OR hazard* OR heavy OR "high risk*" OR problem*))  36,136  **6** TS=(lifestyle* OR life-style*)  161,859  **5** TS=(exercis* OR (physical* NEAR/2 (activ* OR inactiv* OR training)))  655,779  **4** TS=((treatment* OR regimen OR medication* OR patient* ) NEAR/3 (complian* OR adher* OR noncomplian* OR nonadher*))  88,724  **3** TS=(health* NEAR/3 (attitud* OR behavio$r* OR knowledge OR practice*))  153,636  **2** TS=("distance counsel$ing" OR "distance consultation*" OR "e consultation*" OR econsultation* OR "e counsel$ing" OR ecounsel$ing OR "e health*" OR ehealth* OR "e therapies" OR "e therapy" OR etherap* OR "e visit*" OR evisit* OR "m health" OR mhealth OR "mobile counsel$ing" OR "mobile consultation*" OR "remote consultation*" OR "remote counsel$ing" OR telecare OR "tele care" OR "tele health*" OR telehealth* OR "tele consultation*" OR teleconsultation* OR "tele medicine" OR telemedicine OR "tele nurs*" OR telenurs* OR "tele rehabilitation" OR telerehabilitation)  52,570  **1** TS=((home* OR symptom* OR "side effect*") NEAR/2 (manag* OR monitor* OR telemonitor*))  38,345 |
| --- |

**4. PsycInfo**

| \| **#** \| **Query** \| \| --- \| --- \| \| S34 \| S1 AND S30 AND S33 \| \| S33 \| S31 OR S32 \| \| S32 \| TI ( (nurse* OR nursing OR telenurs*) ) OR AB ( (nurse* OR nursing OR telenurs*) ) OR KW ( (nurse* OR nursing OR telenurs*) ) \| \| S31 \| DE "Nursing" OR DE "Nurses" OR DE "Psychiatric Nurses" OR DE "Public Health Service Nurses" OR DE "School Nurses" \| \| S30 \| S2 OR S3 OR S4 OR S5 OR S6 OR S7 OR S8 OR S9 OR S10 OR S11 OR S12 OR S13 OR S14 OR S15 OR S16 OR S17 OR S18 OR S19 OR S20 OR S21 OR S22 OR S23 OR S24 OR S25 OR S26 OR S27 OR S28 OR S29 \| \| S29 \| TI ( (annual* N3 (vaccination* OR immunization* OR immunisation* OR shot#)) ) OR AB ( (annual* N3 (vaccination* OR immunization* OR immunisation* OR shot#)) ) OR KW ( (annual* N3 (vaccination* OR immunization* OR immunisation* OR shot#)) ) \| \| S28 \| DE "Treatment Refusal" OR DE "Vaccination Attitudes" \| \| S27 \| TI ( (anti-smoking OR antismoking OR cigar* OR cigarette* OR nicotine OR smoking OR smoker# OR snuff OR snus OR tobacco) ) OR AB ( (anti-smoking OR antismoking OR cigar* OR cigarette* OR nicotine OR smoking OR smoker# OR snuff OR snus OR tobacco) ) OR KW ( (anti-smoking OR antismoking OR cigar* OR cigarette* OR nicotine OR smoking OR smoker# OR snuff OR snus OR tobacco) ) \| \| S26 \| DE "Smoking Cessation" OR DE "Tobacco Use Disorder" OR DE "Tobacco Smoking" \| \| S25 \| TI ( ((daily N1 weigh*) OR (weight N2 (monitor* OR loss OR reduct*))) ) OR AB ( ((daily N1 weigh*) OR (weight N2 (monitor* OR loss OR reduct*))) ) OR KW ( ((daily N1 weigh*) OR (weight N2 (monitor* OR loss OR reduct*))) ) \| \| S24 \| TI ( ((calori* OR fruit* OR fat* OR fiber* OR fibre* OR portion* OR salt OR sodium OR sugar* OR vegetable*) N3 (choice* OR consum* OR decreas* OR diet OR discourage* OR eat* OR frequenc* OR intake OR increase* OR limit* OR less OR lessen OR number* OR portion* OR preference* OR reduc* OR restriction* OR serving* OR size*)) ) OR AB ( ((calori* OR fruit* OR fat* OR fiber* OR fibre* OR portion* OR salt OR sodium OR sugar* OR vegetable*) N3 (choice* OR consum* OR decreas* OR diet OR discourage* OR eat* OR frequenc* OR intake OR increase* OR limit* OR less OR lessen OR number* OR portion* OR preference* OR reduc* OR restriction* OR serving* OR size*)) ) OR KW ( ((calori* OR fruit* OR fat* OR fiber* OR fibre* OR portion* OR salt OR sodium OR sugar* OR vegetable*) N3 (choice* OR consum* OR decreas* OR diet OR discourage* OR eat* OR frequenc* OR intake OR increase* OR limit* OR less OR lessen OR number* OR portion* OR preference* OR reduc* OR restriction* OR serving* OR size*)) ) \| \| S23 \| TI ( ((food# OR diet*) N3 (choice* OR frequenc* OR intake* OR intervention* OR modification* OR therap*)) ) OR AB ( ((food# OR diet*) N3 (choice* OR frequenc* OR intake* OR intervention* OR modification* OR therap*)) ) OR KW ( ((food# OR diet*) N3 (choice* OR frequenc* OR intake* OR intervention* OR modification* OR therap*)) ) \| \| S22 \| TI ( ((eat* OR diet* OR food# OR feed*) N3 (behavio#r* OR habit* OR pattern*)) ) OR AB ( ((eat* OR diet* OR food# OR feed*) N3 (behavio#r* OR habit* OR pattern*)) ) OR KW ( ((eat* OR diet* OR food# OR feed*) N3 (behavio#r* OR habit* OR pattern*)) ) \| \| S21 \| TI ( ((health* OR unhealth*) N2 (diet* OR eat* OR food* OR habit*)) ) OR AB ( ((health* OR unhealth*) N2 (diet* OR eat* OR food* OR habit*)) ) OR KW ( ((health* OR unhealth*) N2 (diet* OR eat* OR food* OR habit*)) ) \| \| S20 \| DE "Diets" OR DE "Weight Control" OR DE "Eating Behavior" OR DE "Healthy Eating" OR DE "Body Weight" \| \| S19 \| TI temperance* OR AB temperance* OR KW temperance* \| \| S18 \| TI ( (alcohol* N1 (use* OR using)) ) OR AB ( (alcohol* N1 (use* OR using)) ) OR KW ( (alcohol* N1 (use* OR using)) ) \| \| S17 \| TI ( ((alcohol* OR binge*) N3 (addict* OR abus* OR abstinen* OR consumption* OR dependen* OR drink* OR intake* OR misus* OR problem* OR rehabilit* OR restrict*)) ) OR AB ( ((alcohol* OR binge*) N3 (addict* OR abus* OR abstinen* OR consumption* OR dependen* OR drink* OR intake* OR misus* OR problem* OR rehabilit* OR restrict*)) ) OR KW ( ((alcohol* OR binge*) N3 (addict* OR abus* OR abstinen* OR consumption* OR dependen* OR drink* OR intake* OR misus* OR problem* OR rehabilit* OR restrict*)) ) \| \| S16 \| TI ( (drink* N2 (behavio#r* OR binge* OR excessive* OR harm* OR hazard* OR heavy OR "high risk*" OR problem*)) ) OR AB ( (drink* N2 (behavio#r* OR binge* OR excessive* OR harm* OR hazard* OR heavy OR "high risk*" OR problem*)) ) OR KW ( (drink* N2 (behavio#r* OR binge* OR excessive* OR harm* OR hazard* OR heavy OR "high risk*" OR problem*)) ) \| \| S15 \| DE "Drinking Behavior" OR DE "Alcohol Drinking Patterns" OR DE "Sobriety" OR DE "Binge Drinking" \| \| S14 \| TI ( (lifestyle* OR life-style*) ) OR AB ( (lifestyle* OR life-style*) ) OR KW ( (lifestyle* OR life-style*) ) \| \| S13 \| DE "Lifestyle" OR DE "Lifestyle Changes" \| \| S12 \| TI ( (exercis* OR (physical* N2 (activ* OR inactiv* OR training))) ) OR AB ( (exercis* OR (physical* N2 (activ* OR inactiv* OR training))) ) OR KW ( (exercis* OR (physical* N2 (activ* OR inactiv* OR training))) ) \| \| S11 \| DE "Exercise" \| \| S10 \| TI ( ((treatment* OR regimen OR medication* OR patient*) N3 (complian* OR adher* OR noncomplian* OR nonadher*)) ) OR AB ( ((treatment* OR regimen OR medication* OR patient*) N3 (complian* OR adher* OR noncomplian* OR nonadher*)) ) OR KW ( ((treatment* OR regimen OR medication* OR patient*) N3 (complian* OR adher* OR noncomplian* OR nonadher*)) ) \| \| S9 \| DE "Treatment Compliance" \| \| S8 \| TI ( (health* N3 (attitud* OR behavio#r* OR knowledge OR practice*)) ) OR AB ( (health* N3 (attitud* OR behavio#r* OR knowledge OR practice*)) ) OR KW ( (health* N3 (attitud* OR behavio#r* OR knowledge OR practice*)) ) \| \| S7 \| DE "Health Knowledge" OR DE "Health Attitudes" OR DE "Health Behavior" \| \| S6 \| TI ( ("distance counsel#ing" OR "distance consultation*" OR "e consultation*" OR econsultation* OR "e counsel#ing" OR ecounsel#ing OR "e health*" OR ehealth* OR "e therapies" OR "e therapy" OR etherap* OR "e visit*" OR evisit* OR "m health" OR mhealth OR "mobile counsel#ing" OR "mobile consultation*" OR "remote consultation*" OR "remote counsel#ing" OR telecare OR "tele care" OR "tele health*" OR telehealth* OR "tele consultation*" OR teleconsultation* OR "tele medicine" OR telemedicine OR "tele nurs*" OR telenurs* OR "tele rehabilitation" OR telerehabilitation) ) OR AB ( ("distance counsel#ing" OR "distance consultation*" OR "e consultation*" OR econsultation* OR "e counsel#ing" OR ecounsel#ing OR "e health*" OR ehealth* OR "e therapies" OR "e therapy" OR etherap* OR "e visit*" OR evisit* OR "m health" OR mhealth OR "mobile counsel#ing" OR "mobile consultation*" OR "remote consultation*" OR "remote counsel#ing" OR telecare OR "tele care" OR "tele health*" OR telehealth* OR "tele consultation*" OR teleconsultation* OR "tele medicine" OR telemedicine OR "tele nurs*" OR telenurs* OR "tele rehabilitation" OR telerehabilitation) ) OR KW ( ("distance counsel#ing" OR "distance consultation*" OR "e consultation*" OR econsultation* OR "e counsel#ing" OR ecounsel#ing OR "e health*" OR ehealth* OR "e therapies" OR "e therapy" OR etherap* OR "e visit*" OR evisit* OR "m health" OR mhealth OR "mobile counsel#ing" OR "mobile consultation*" OR "remote consultation*" OR "remote counsel#ing" OR telecare OR "tele care" OR "tele health*" OR telehealth* OR "tele consultation*" OR teleconsultation* OR "tele medicine" OR telemedicine OR "tele nurs*" OR telenurs* OR "tele rehabilitation" OR telerehabilitation) ) \| \| S5 \| DE "Telemedicine" OR DE "Online Therapy" OR DE "Teleconsultation" OR DE "Telerehabilitation" \| \| S4 \| TI ( ((home* OR symptom* OR "side effect*") N2 (manag* OR monitor* OR telemonitor*)) ) OR AB ( ((home* OR symptom* OR "side effect*") N2 (manag* OR monitor* OR telemonitor*)) ) OR KW ( ((home* OR symptom* OR "side effect*") N2 (manag* OR monitor* OR telemonitor*)) ) \| \| S3 \| TI ( (selfadministrat* OR self-administrat* OR selfcare OR self-care OR self-caring OR self-examination OR selfexamination OR selfhelp* OR self-help OR selfmanag* OR self-manag* OR self-monitor* OR selftreat* OR self-treat*) ) OR AB ( (selfadministrat* OR self-administrat* OR selfcare OR self-care OR self-caring OR self-examination OR selfexamination OR selfhelp* OR self-help OR selfmanag* OR self-manag* OR self-monitor* OR selftreat* OR self-treat*) ) OR KW ( (selfadministrat* OR self-administrat* OR selfcare OR self-care OR self-caring OR self-examination OR selfexamination OR selfhelp* OR self-help OR selfmanag* OR self-manag* OR self-monitor* OR selftreat* OR self-treat*) ) \| \| S2 \| DE "Self-Management" OR DE "Self-Care" OR DE "Self-Monitoring" \| \| S1 \| TI ( ((cardiac OR heart OR myocardi* OR ventric*) N3 (decompensat* OR dysfunction* OR insufficien* OR failure)) ) OR AB ( ((cardiac OR heart OR myocardi* OR ventric*) N3 (decompensat* OR dysfunction* OR insufficien* OR failure)) ) OR KW ( ((cardiac OR heart OR myocardi* OR ventric*) N3 (decompensat* OR dysfunction* OR insufficien* OR failure)) ) \| |
| --- | --- | --- | --- | --- | --- | --- | --- | --- | --- | --- | --- | --- | --- | --- | --- | --- | --- | --- | --- | --- | --- | --- | --- | --- | --- | --- | --- | --- | --- | --- | --- | --- | --- | --- | --- | --- | --- | --- | --- | --- | --- | --- | --- | --- | --- | --- | --- | --- | --- | --- | --- | --- | --- | --- | --- | --- | --- | --- | --- | --- | --- | --- | --- | --- | --- | --- | --- | --- | --- | --- |

**5. Cinahl**

| \| **#** \| **Query** \| \| --- \| --- \| \| S37 \| S31 AND S32 AND S36 \| \| S36 \| S33 OR S34 OR S35 \| \| S35 \| TI ( (nurse* OR nursing OR telenurs*) ) OR AB ( (nurse* OR nursing OR telenurs*) ) \| \| S34 \| (MH "Nurses+") OR (MH "Nursing Care+") OR (MH "Nursing Role") \| \| S33 \| MW Nursing \| \| S32 \| S3 OR S4 OR S5 OR S6 OR S7 OR S8 OR S9 OR S10 OR S11 OR S12 OR S13 OR S14 OR S15 OR S16 OR S17 OR S18 OR S19 OR S20 OR S21 OR S22 OR S23 OR S24 OR S25 OR S26 OR S27 OR S28 OR S29 OR S30 \| \| S31 \| S1 OR S2 \| \| S30 \| TI ( (annual* N3 (vaccination* OR immunization* OR immunisation* OR shot#)) ) OR AB ( (annual* N3 (vaccination* OR immunization* OR immunisation* OR shot#)) ) \| \| S29 \| (MH "Treatment Refusal") \| \| S28 \| TI ( (anti-smoking OR antismoking OR cigar* OR cigarette* OR nicotine OR smoking OR smoker# OR snuff OR snus OR tobacco) ) OR AB ( (anti-smoking OR antismoking OR cigar* OR cigarette* OR nicotine OR smoking OR smoker# OR snuff OR snus OR tobacco) ) \| \| S27 \| (MH "Smoking Cessation") OR (MH "Smoking+") \| \| S26 \| TI ( ((daily N1 weigh*) OR (weight N2 (monitor* OR loss OR reduct*))) ) OR AB ( ((daily N1 weigh*) OR (weight N2 (monitor* OR loss OR reduct*))) ) \| \| S25 \| TI ( ((calori* OR fruit* OR fat* OR fiber* OR fibre* OR portion* OR salt OR sodium OR sugar* OR vegetable*) N3 (choice* OR consum* OR decreas* OR diet OR discourage* OR eat* OR frequenc* OR intake OR increase* OR limit* OR less OR lessen OR number* OR portion* OR preference* OR reduc* OR restriction* OR serving* OR size*)) ) OR AB ( ((calori* OR fruit* OR fat* OR fiber* OR fibre* OR portion* OR salt OR sodium OR sugar* OR vegetable*) N3 (choice* OR consum* OR decreas* OR diet OR discourage* OR eat* OR frequenc* OR intake OR increase* OR limit* OR less OR lessen OR number* OR portion* OR preference* OR reduc* OR restriction* OR serving* OR size*)) ) \| \| S24 \| TI ( ((food# OR diet*) N3 (choice* OR frequenc* OR intake* OR intervention* OR modification* OR therap*)) ) OR AB ( ((food# OR diet*) N3 (choice* OR frequenc* OR intake* OR intervention* OR modification* OR therap*)) ) \| \| S23 \| TI ( ((eat* OR diet* OR food# OR feed*) N3 (behavio#r* OR habit* OR pattern*)) ) OR AB ( ((eat* OR diet* OR food# OR feed*) N3 (behavio#r* OR habit* OR pattern*)) ) \| \| S22 \| TI ( ((health* OR unhealth*) N2 (diet* OR eat* OR food* OR habit*)) ) OR AB ( ((health* OR unhealth*) N2 (diet* OR eat* OR food* OR habit*)) ) \| \| S21 \| (MH "Diet+") OR (MH "Eating Behavior") OR (MH "Weight Reduction Programs") OR (MH "Body Weight+") OR (MH "Food Habits") \| \| S20 \| TI temperance* OR AB temperance* \| \| S19 \| TI ( (alcohol* N1 (use* OR using)) ) OR AB ( (alcohol* N1 (use* OR using)) ) \| \| S18 \| TI ( ((alcohol* OR binge*) N3 (addict* OR abus* OR abstinen* OR consumption* OR dependen* OR drink* OR intake* OR misus* OR problem* OR rehabilit* OR restrict*)) ) OR AB ( ((alcohol* OR binge*) N3 (addict* OR abus* OR abstinen* OR consumption* OR dependen* OR drink* OR intake* OR misus* OR problem* OR rehabilit* OR restrict*)) ) \| \| S17 \| TI ( (drink* N2 (behavio#r* OR binge* OR excessive* OR harm* OR hazard* OR heavy OR "high risk*" OR problem*)) ) OR AB ( (drink* N2 (behavio#r* OR binge* OR excessive* OR harm* OR hazard* OR heavy OR "high risk*" OR problem*)) ) \| \| S16 \| (MH "Drinking Behavior+") \| \| S15 \| TI ( (lifestyle* OR life-style*) ) OR AB ( (lifestyle* OR life-style*) ) \| \| S14 \| (MH "Life Style Changes") OR (MH "Life Style") \| \| S13 \| TI ( (exercis* OR (physical* N2 (activ* OR inactiv* OR training))) ) OR AB ( (exercis* OR (physical* N2 (activ* OR inactiv* OR training))) ) \| \| S12 \| (MH "Exercise") \| \| S11 \| TI ( ((treatment* OR regimen OR medication* OR patient*) N3 (complian* OR adher* OR noncomplian* OR nonadher*)) ) OR AB ( ((treatment* OR regimen OR medication* OR patient*) N3 (complian* OR adher* OR noncomplian* OR nonadher*)) ) \| \| S10 \| (MH "Patient Compliance+") \| \| S9 \| TI ( (health* N3 (attitud* OR behavio#r* OR knowledge OR practice*)) ) OR AB ( (health* N3 (attitud* OR behavio#r* OR knowledge OR practice*)) ) \| \| S8 \| (MH "Health Knowledge") OR (MH "Attitude to Health") OR (MH "Health Behavior") \| \| S7 \| TI ( ("distance counsel#ing" OR "distance consultation*" OR "e consultation*" OR econsultation* OR "e counsel#ing" OR ecounsel#ing OR "e health*" OR ehealth* OR "e therapies" OR "e therapy" OR etherap* OR "e visit*" OR evisit* OR "m health" OR mhealth OR "mobile counsel#ing" OR "mobile consultation*" OR "remote consultation*" OR "remote counsel#ing" OR telecare OR "tele care" OR "tele health*" OR telehealth* OR "tele consultation*" OR teleconsultation* OR "tele medicine" OR telemedicine OR "tele nurs*" OR telenurs* OR "tele rehabilitation" OR telerehabilitation) ) OR AB ( ("distance counsel#ing" OR "distance consultation*" OR "e consultation*" OR econsultation* OR "e counsel#ing" OR ecounsel#ing OR "e health*" OR ehealth* OR "e therapies" OR "e therapy" OR etherap* OR "e visit*" OR evisit* OR "m health" OR mhealth OR "mobile counsel#ing" OR "mobile consultation*" OR "remote consultation*" OR "remote counsel#ing" OR telecare OR "tele care" OR "tele health*" OR telehealth* OR "tele consultation*" OR teleconsultation* OR "tele medicine" OR telemedicine OR "tele nurs*" OR telenurs* OR "tele rehabilitation" OR telerehabilitation) ) \| \| S6 \| (MH "Telemedicine+") OR (MH "Telenursing") \| \| S5 \| TI ( ((home* OR symptom* OR "side effect*") N2 (manag* OR monitor* OR telemonitor*)) ) OR AB ( ((home* OR symptom* OR "side effect*") N2 (manag* OR monitor* OR telemonitor*)) ) \| \| S4 \| TI ( (selfadministrat* OR self-administrat* OR selfcare OR self-care OR self-caring OR self-examination OR selfexamination OR selfhelp* OR self-help OR selfmanag* OR self-manag* OR self-monitor* OR selftreat* OR self-treat*) ) OR AB ( (selfadministrat* OR self-administrat* OR selfcare OR self-care OR self-caring OR self-examination OR selfexamination OR selfhelp* OR self-help OR selfmanag* OR self-manag* OR self-monitor* OR selftreat* OR self-treat*) ) \| \| S3 \| (MH "Self Care+") \| \| S2 \| TI ( ((cardiac OR heart OR myocardi* OR ventric*) N3 (decompensat* OR dysfunction* OR insufficien* OR failure)) ) OR AB ( ((cardiac OR heart OR myocardi* OR ventric*) N3 (decompensat* OR dysfunction* OR insufficien* OR failure)) ) \| \| S1 \| (MH "Heart Failure+") \| |
| --- | --- | --- | --- | --- | --- | --- | --- | --- | --- | --- | --- | --- | --- | --- | --- | --- | --- | --- | --- | --- | --- | --- | --- | --- | --- | --- | --- | --- | --- | --- | --- | --- | --- | --- | --- | --- | --- | --- | --- | --- | --- | --- | --- | --- | --- | --- | --- | --- | --- | --- | --- | --- | --- | --- | --- | --- | --- | --- | --- | --- | --- | --- | --- | --- | --- | --- | --- | --- | --- | --- | --- | --- | --- | --- | --- | --- |

# Additional file 3. List of excluded studies from the full-text screening and the main reason of exclusion

**Setting not appropriate to satisfy inclusion criteria**

1. Agren S, Evangelista LS, Hjelm C, Stromberg A. Dyads affected by chronic heart failure: a randomized study evaluating effects of education and psychosocial support to patients with heart failure and their partners. Journal of Cardiac Failure. 2012;18(5):359-66.
2. Arruda CS, Pereira JMV, Figueiredo LDS, Scofano BDS, Flores PVP, Cavalcanti ACD. Effect of an orientation group for patients with chronic heart failure: randomized controlled trial. Revista Latino-Americana de Enfermagem. 2018;25:e2982.
3. Boyne JJ, Vrijhoef HJ, Spreeuwenberg M, De Weerd G, Kragten J, Gorgels AP. Effects of tailored telemonitoring on heart failure patients' knowledge, self-care, self-efficacy and adherence: a randomized controlled trial. European journal of cardiovascular nursing. 2014;13(3):243‐52.
4. Chen HM, Wang ST, Wu SJ, Lee CS, Fetzer SJ, Tsai LM. Effects of Predischarge Patient Education Combined With Postdischarge Follow-Ups on Self-Care, Readmission, Sleep, and Depression in Patients With Heart Failure. Journal of Nursing Research. 2020;28(5):e112.
5. Chen SH, Boyd J, all S, Maiorana A. Association between community-based nurse practitioner support, self-care behaviour and quality of life in patients with chronic heart failure. Australian Journal of Advanced Nursing. 2021;38(3):25-32.
6. Chew HSJ, Sim KLD, Choi KC, Chair SY. Effectiveness of a nurse-led temporal self-regulation theory-based program on heart failure self-care: A randomized controlled trial (vol 115, 103872, 2021). International Journal of Nursing Studies. 2021;121:1.
7. Cockayne S, Pattenden J, Worthy G, Richardson G, Lewin R. Nurse facilitated Self-management support for people with heart failure and their family carers (SEMAPHFOR): a randomised controlled trial. International Journal of Nursing Studies. 2014;51(9):1207-13.
8. Cui X, Zhou X, Ma LL, Sun TW, Bishop L, Gardiner FW, et al. A nurse-led structured education program improves self-management skills and reduces hospital readmissions in patients with chronic heart failure: a randomized and controlled trial in China. Rural & Remote Health. 2019;19(2):5270.
9. de la Porte PW, Lok DJ, van Veldhuisen DJ, van Wijngaarden J, Cornel JH, Zuithoff NP, et al. Added value of a physician-and-nurse-directed heart failure clinic: results from the Deventer-Alkmaar heart failure study. Heart. 2007;93(7):819-25.
10. Flores PVP, Rocha PA, Figueiredo LD, Guimaraes TML, Velasco NS, Cavalcanti ACD. Effect of motivational interviewing on self-care of people with heart failure: a randomized clinical trial. Revista Da Escola De Enfermagem Da Usp. 2020;54:8.
11. Garcimartin P, Astals-Vizcaino M, Badosa N, Linas A, Ivern C, Duran X, et al. The Impact of Motivational Interviewing on Self-care and Health-Related Quality of Life in Patients With Chronic Heart Failure. Journal of Cardiovascular Nursing. 2021;7:07.
12. Granger BB, Bosworth H, Hern, ez A, Reed S, Ekman I. Results of the chronic heart failure intervention to improve medication adherence (chime) study: a randomized self-management intervention in high risk non-adherent patients. Circulation. 2013;128(22).
13. Granger BB, Ekman I, Hern, ez AF, Sawyer T, Bowers MT, et al. Results of the Chronic Heart Failure Intervention to Improve MEdication Adherence study: A randomized intervention in high-risk patients. American Heart Journal. 2015;169(4):539-48.
14. Howie-Esquivel J, Bibbins-Domingo K, Clark R, Evangelista L, Dracup K. A Culturally Appropriate Educational Intervention Can Improve Self-Care in Hispanic Patients With Heart Failure: A Pilot Randomized Controlled Trial. Cardiology Research. 2014;5(3):91-100.
15. Mizukawa M, Moriyama M, Yamamoto H, Rahman MM, Naka M, Kitagawa T, et al. Nurse-Led Collaborative Management Using Telemonitoring Improves Quality of Life and Prevention of Rehospitalization in Patients with Heart Failure. International heart journal. 2019;60(6):1293‐302.
16. Moghadam FM, Naderi N, Shabani F, Taghavi S, Mousavizadeh R, Fathollahi MS. Effects of Spiritual Care Program on Quality of Life in Patients with Heart Failure. Journal of Evidence-based Care. 2021;11(2):44-53.
17. Oscalices MIL, Okuno MFP, Lopes M, Campanharo CRV, Batista REA. Discharge guidance and telephone follow-up in the therapeutic adherence of heart failure: randomized clinical trial. Revista Latino-Americana De Enfermagem. 2019;27:9.
18. Ostergaard B, Mahrer-Imhof R, Wagner L, Barington T, Videbaek L, Lauridsen J. Effect of family nursing therapeutic conversations on health-related quality of life, self-care and depression among outpatients with heart failure: A randomized multi-centre trial. Patient Education & Counseling. 2018;101(8):1385-93.
19. Otsu H, Moriyama M. Effectiveness of an educational self-management program for outpatients with chronic heart failure. Japan Journal of Nursing Science: JJNS. 2011;8(2):140-52.
20. Paradis V, Cossette S, Frasure-Smith N, Heppell S, Guertin MC. The efficacy of a motivational nursing intervention based on the stages of change on self-care in heart failure patients. Journal of Cardiovascular Nursing. 2010;25(2):130-41.
21. Pereira Sousa J, Neves H, Pais-Vieira M. Does Symptom Recognition Improve Self-Care in Patients with Heart Failure? A Pilot Study Randomised Controlled Trial. Nursing Reports. 2021;11(2):418-29.
22. Rivas AH, ez, Gomez TS, Rodriguez MM, Villanueva NA, Diez MN, et al. Evaluation of the implementation of an educational intervention nurse in patients treated at the Heart Failure Unit at the University Hospital Puerta de Hierro. Nure Investigacion. 2012(61):9p-p.
23. Rodriguez-Gazquez L, Arredondo-Holguin E, Herrera-Cortes R. Effectiveness of an educational program in nursing in the self-care of patients with heart failure: randomized controlled trial. Revista Latino-Americana de Enfermagem. 2012;20(2):296-306.
24. Sezgin D, Mert H, Ozpelit E, Akdeniz B. The effect on patient outcomes of a nursing care and follow-up program for patients with heart failure: A randomized controlled trial. International Journal of Nursing Studies. 2017;70:17-26.
25. Shearer NB, Cisar N, Greenberg EA. A telephone-delivered empowerment intervention with patients diagnosed with heart failure. Heart & Lung. 2007;36(3):159-69.
26. Smeulders ES, van Haastregt JC, Ambergen T, Uszko-Lencer NH, Janssen-Boyne JJ, Gorgels AP, et al. Nurse-led self-management group programme for patients with congestive heart failure: randomized controlled trial. Journal of Advanced Nursing. 2010;66(7):1487-99.
27. Smith CE, Piamjariyakul U, Dalton KM, Russell C, Wick J, Ellerbeck EF. Nurse-Led Multidisciplinary Heart Failure Group Clinic Appointments: Methods, Materials, and Outcomes Used in the Clinical Trial. Journal of Cardiovascular Nursing. 2015;30(4):S25-34.
28. Srisuk N, Cameron J, Ski CF, Thompson DR. A family-based education program for heart failure patients and carers in rural Thailand: a randomised controlled trial. Heart lung and circulation. 2015;24:S418‐.
29. Srisuk N, Cameron J, Ski CF, Thompson DR. Randomized controlled trial of family-based education for patients with heart failure and their carers. Journal of Advanced Nursing. 2017;73(4):857-70.
30. Stamp KD, Dunbar SB, Clark PC, Reilly CM, Gary RA, Higgins M, et al. Family partner intervention influences self-care confidence and treatment self-regulation in patients with heart failure. European Journal of Cardiovascular Nursing. 2016;15(5):317-27.
31. Stromberg A, Martensson J, Fridlund B, Levin LA, Karlsson JE, Dahlstrom U. Nurse-led heart failure clinics improve survival and self-care behaviour in patients with heart failure: results from a prospective, randomised trial. European Heart Journal. 2003;24(11):1014-23.
32. Sun J, Zhang ZW, Ma YX, Liu W, Wang CY. Application of self-care based on full-course individualized health education in patients with chronic heart failure and its influencing factors. World Journal of Clinical Cases. 2019;7(16):2165-75.
33. Tawalbeh LI. The Effect of Cardiac Education on Knowledge and Self-care Behaviors Among Patients With Heart Failure. Dimensions of Critical Care Nursing. 2018;37(2):78-86.
34. Wang ZR, Zhou JW, Liu XP, Cai GJ, Zhang QH, Mao JF. Effects of WeChat platform-based health management on health and self-management effectiveness of patients with severe chronic heart failure. World Journal of Clinical Cases. 2021;9(34):10576-84.
35. Wierzchowiecki M, Poprawski K, Nowicka A, ziora M, Piatkowska A, Jankowiak M, et al. A new programme of multidisciplinary care for patients with heart failure in Poznań: one-year follow-up. Kardiologia polska. 2006;64(10):1063‐70; discussion 71‐2.
36. Yehle KS, s LP, Rhynders PA, Newton GD. The effect of shared medical visits on knowledge and self-care in patients with heart failure: a pilot study. Heart & Lung. 2009;38(1):25-33.
37. Yu H, Zhang P, Wang X, Wang Y, Zhang B. Effect of Health Education Based on Behavioral Change Theories on Self-Efficacy and Self-Management Behaviors in Patients with Chronic Heart Failure. Iranian Journal of Public Health. 2019;48(3):421-8.

**Outcome not appropriate to satisfy inclusion criteria**

1. Benatar D, Bondmass M, Ghitelman J, Avitall B. Outcomes of chronic heart failure. Archives of Internal Medicine. 2003;163(3):347-52.
2. Ching Yu L, Chia Yuan L, Shu Nu Chang L, Wen Chun L, Yu Ju C. The Effectiveness of Case Management for Patients with Heart Failure. Macau Journal of Nursing. 2013;12(1):35-41.
3. Domingues FB, Clausell N, Aliti GB, Dominguez DR, Rabelo ER. Education and telephone monitoring by nurses of patients with heart failure: randomized clinical trial. Arquivos Brasileiros de Cardiologia. 2011;96(3):233-9.
4. Duncan K, Pozehl B. Effects of an exercise adherence intervention on outcomes in patients with heart failure. Rehabilitation Nursing Journal. 2003;28(4):117-22.
5. Duncan K, Pozehl B, Hertzog M, Norman JF. Psychological responses and adherence to exercise in heart failure. Rehabilitation Nursing Journal. 2014;39(3):130-9.
6. Gary R. Exercise self-efficacy in older women with diastolic heart failure: results of a walking program and education intervention. Journal of Gerontological Nursing. 2006;32(7):31-9; quiz 40-31.
7. Johnston B, Wheeler L, Deuser J, Sousa KH. Outcomes of the Kaiser Permanente Tele-Home Health Research Project. Archives of Family Medicine. 2000;9(1):40-5.
8. Karlsson MR, Edner M, Henriksson P, Mejhert M, Persson H, Grut M, et al. A nurse-based management program in heart failure patients affects females and persons with cognitive dysfunction most. Patient Education & Counseling. 2005;58(2):146-53.
9. Kutzleb J, Reiner D. The impact of nurse-directed patient education on quality of life and functional capacity in people with heart failure. Journal of the American Academy of Nurse Practitioners. 2006;18(3):116-23.
10. LaFramboise LM, Todero CM, Zimmerman L, Agrawal S, LaFramboise LM, Todero CM, et al. Comparison of Health Buddy with traditional approaches to heart failure management. Family & Community Health. 2003;26(4):275-88.
11. Padula CA, Yeaw E, Mistry S. A home-based nurse-coached inspiratory muscle training intervention in heart failure. Applied Nursing Research. 2009;22(1):18-25.
12. Pressler SJ, Therrien B, Riley PL, Chou CC, Ronis DL, Koelling TM, et al. Nurse-Enhanced Memory Intervention in Heart Failure: the MEMOIR study. Journal of Cardiac Failure. 2011;17(10):832-43.
13. Rakhshan M, Kordshooli KR, Ghadakpoor S. Effects of Family-Center Empowerment Model on the Lifestyle of Heart Failure Patients: A Randomized Controlled Clinical Trial. International Journal of Community Based Nursing & Midwifery. 2015;3(4):255-62.
14. Rimoldi A, Raimondo R, Bortolan L, Braga SS. Medium- long term effectiveness of nursing management in a telecare program for patients with medium-high degree heart failure. European Heart Journal Supplements. 2020;22:G176-G.
15. Stamp KD, Dunbar SB, Clark PC, Reilly CM, Gary RA, Higgins M, et al. Family partner intervention influences self-care confidence and treatment self-regulation in patients with heart failure. European Journal of Cardiovascular Nursing. 2016;15(5):317-27.
16. Tiozzo SN, Basso C, Capodaglio G, Schievano E, Dotto M, Avossa F, et al. Effectiveness of a community care management program for multimorbid elderly patients with heart failure in the Veneto Region. Aging-Clinical & Experimental Research. 2019;31(2):241-7.
17. Wakefield, B. J., Holman, J. E., Ray, A., Scherubel, M., Burns, T. L., Kienzle, M. G., & Rosenthal, G. E. (2009). Outcomes of a home telehealth intervention for patients with heart failure. Journal of telemedicine and telecare, 15(1), 46–50. https://doi.org/10.1258/jtt.2008.080701
18. White M, Garbez R, Carroll M, Brinker E, Howie-Esquivel J. Is "teach-back" associated with knowledge retention and hospital readmission in hospitalized heart failure patients? Journal of Cardiovascular Nursing. 2013;28(2):137-46.
19. You J, Wang S, Li J, Luo Y. Usefulness of a Nurse-Led Program of Care for Management of Patients with Chronic Heart Failure. Medical Science Monitor. 2020;26:e920469.

**Study design not appropriate to satisfy inclusion criteria**

1. Bryant, R., & Himawan, L. (2019). Heart Failure Self-care Program Effect on Outcomes. JNP-JOURNAL FOR NURSE PRACTITIONERS, 15(5), 379-381.
2. Enç, N., Yigit, Z., & Altiok, M. G. (2010). Effects of education on self-care behaviour and quality of life in patients with chronic heart failure. CONNECT: The World of Critical Care Nursing, 7(2), 115-121.
3. Ensign, C. M., & Hawkins, S. Y. (2017). Improving Patient Self-Care and Reducing Readmissions Through an Outpatient Heart Failure Case Management Program. Professional Case Management, 22(4), 190-196.
4. Khankaew, K., Ongsombat, N., Wonginchan, A., Senthong, V., & Thronsao, C. (2020). Outcomes of Self-Care in Clients with Heart Failure before and after Treatment, using a Case Management Approach. Disability, CBR & Inclusive Development, 31(2), 132-147.
5. Garcimartin, P., Astals-Vizcaino, M., Badosa, N., Linas, A., Ivern, C., Duran, X., & Comin-Colet, J. (2021). The Impact of Motivational Interviewing on Self-care and Health-Related Quality of Life in Patients With Chronic Heart Failure. Journal of Cardiovascular Nursing, 7, 07.
6. Law, T., Jones, S., & Vardaman, S. (2019). Implementation of a Shared Medical Appointment as a Holistic Approach to CHF Management. Holistic nursing practice, 33(6), 354-359.
7. Ryan, M., Aloe, K., & Mason-Johnson, J. (2009). Improving Self-management and Reducing Hospital Readmission in Heart Failure Patients. Clinical Nurse Specialist, 23(4), 216-221.
8. Shively, M. J., Gardetto, N. J., Kodiath, M. F., Kelly, A., Smith, T. L., Stepnowsky, C., Maynard, C., & Larson, C. B. (2013). Effect of Patient Activation on Self-Management in Patients With Heart Failure. Journal of Cardiovascular Nursing, 28(1), 20-34.
9. Smeulders, E., Van Haastregt, J. C. M., Van Hoef, E. F. M., Van Eijk, J., & Kempen, G. (2006). Evaluation of a self-management programme for congestive heart failure patients: design of a randomised controlled trial. BMC Health Services Research, 6.
10. Tawalbeh, L. I. (2018). The Effect of Cardiac Education on Knowledge and Self-care Behaviors Among Patients With Heart Failure. Dimensions of Critical Care Nursing, 37(2), 78-86.
11. West, J. A., Miller, N. H., Parker, K. M., Senneca, D., Gh, our, G., Clark, M., Greenwald, G., Heller, R. S., Fowler, M. B., & DeBusk, R. F. (1997). A comprehensive management system for heart failure improves clinical outcomes and reduces medical resource utilization. American Journal of Cardiology, 79(1), 58-63.

**Intervention not appropriate to satisfy inclusion criteria**

1. Barnason, S., Zimmerman, L., Nieveen, J., Schmaderer, M., Carranza, B., & Reilly, S. (2003). Impact of a home communication intervention for coronary artery bypass graft patients with ischemic heart failure on self-efficacy, coronary disease risk factor modification, and functioning. HEART & LUNG, 32(3), 147-158.
2. Brodie, D. A., & Inoue, A. (2005). Motivational interviewing to promote physical activity for people with chronic heart failure. Journal of Advanced Nursing, 50(5), 518-527.
3. Brodie, D. A., Inoue, A., & Shaw, D. G. (2008). Motivational interviewing to change quality of life for people with chronic heart failure: a randomised controlled trial. International Journal of Nursing Studies, 45(4), 489-500.
4. Freedl, K. E., Carney, R. M., Rich, M. W., Steinmeyer, B. C., & Rubin, E. H. (2015). Cognitive Behavior Therapy for Depression and Self-Care in Heart Failure Patients: A Randomized Clinical Trial. JAMA INTERNAL MEDICINE, 175(11), 1773-1782.
5. Sarboozi Hosein َAbadi, T., Namazi Nia, M., & Mazlom, S. R. (2020). Effect of Self-determination Theory-based Discharge Program on Lifestyle and Readmission of Patients with Heart Failure: A Clinical Trial. Evidence Based Care, 10(1), 25-35.
6. Wang, Z. R., Zhou, J. W., Liu, X. P., Cai, G. J., Zhang, Q. H., & Mao, J. F. (2021). Effects of WeChat platform-based health management on health and self-management effectiveness of patients with severe chronic heart failure. World Journal of Clinical Cases, 9(34), 10576-10584.

**Population not appropriate to satisfy inclusion criteria**

1. Aiken, L. S., Butner, J., Lockhart, C. A., Volk-Craft, B. E., Hamilton, G., & Williams, F. G. (2006). Outcome evaluation of a randomized trial of the PhoenixCare intervention: program of case management and coordinated care for the seriously chronically ill. Journal of Palliative Medicine, 9(1), 111-126.
2. Zakrisson, A. B., Arne, M., Hasselgren, M., Lisspers, K., Stallberg, B., The, & er, K. (2019). A complex intervention of self-management for patients with COPD or CHF in primary care improved performance and satisfaction with regard to own selected activities; A longitudinal follow-up. Journal of Advanced Nursing, 75(1), 175-186.

**Language not appropriate to satisfy inclusion criteria**

1. Shim, J. L., & Hwang, S. Y. (2016). Development and Effects of a Heart Health Diary for Self-Care Enhancement of Patients with Heart Failure. Journal of Korean Academy of Nursing, 46(6), 881-893.
2. زهرا, ف., مسعود, چ., آرمین, ز., & فاطمه, س. (2019). بررسی تأثیر برنامه مراقبتی مبتنیبر مدل پندر بر رفتارهاي ارتقادهنده سلامتو خود مراقبتیبیماران مبتلا به نارساییقلبی: کارآزمایی کنترل شده تصادفی یک سوکور. HAYAT, 25(2), 106-123.
